# Hybrid Laparoscopic Versus Open Pancreatoduodenectomy. A Meta-Analysis

**DOI:** 10.1007/s00268-021-06372-1

**Published:** 2022-01-18

**Authors:** Miljana Vladimirov, Dirk Bausch, Hubert J. Stein, Tobias Keck, Ulrich Wellner

**Affiliations:** 1 Klinik für Allgemein, Viszeral- und Thoraxchirurgie, PMU Nürnberg, Nuremberg, Deutschland; 2 Klinik für Chirurgie, Universitätsklinikum Schleswig-Holstein, Campus Lübeck, Ratzeburger Allee 160, 23538 Lübeck, Deutschland

## Abstract

**Introduction:**

Hybrid laparoscopic techniques have been proposed as a good transition from open to complete minimally invasive approach especially in complex surgical procedures. This meta-analysis aimed to compare the outcomes of hybrid laparoscopic pancreatoduodenectomy versus open pancreatoduodenectomy.

**Methods:**

A systematic literature research was performed according to PRISMA guidelines. A broad search strategy with terms “laparoscopy” and “pancreatoduodenectomy” was used. Included studies were analyzed by quantitative meta-analysis using the metafor package for R software.

**Results:**

Of 655 identified articles, 627 were excluded and 28 articles fully assessed, including 14 comparative studies, 8 case series and 6 case reports. Extracted data included intraoperative variables and postoperative outcome parameters. The predefined inclusion criteria were met by 14 comparative studies, and 371 patients were pooled in the meta-analysis. Hybrid laparoscopic pacreatoduodenectomy was associated with significantly longer operative time (*I*^2^ 0%, *p* = 0,01, Mean HPD 494,6 min, Mean OPD 421,6 min, WMD 67 min, 95% CI 14–120 min). For all other postoperative outcome parameters, no statistically significant differences were found. A nonsignificant reduction in intraoperative transfusion rate (*I*^2^ 20%, *p* = 0,2, proportion HPD 2%, proportion OPD 1,6%, OR 0,44, 95% CI 0,16–1,27) and blood loss (*I*^2^ 95%, *p* = 0,1, Mean HPD 397,2 ml, Mean OPD 1017,8 ml, MD − 601 ml, 95% CI − 1311–108) was observed for hybrid pancreatoduodenectomy in comparison to open surgery.

**Conclusions:**

This meta-analysis demonstrates significantly increased operation time for hybrid laparoscopic compared to open pancreatoduodenectomy. Intraoperative variables as well as postoperative parameters and major morbidity were comparable for both techniques. Overall results of this meta-analysis demonstrated the hybrid technique as a safe procedure in high-volume centers offering aspects of a safe transition to fully laparoscopic pancreatoduodenectomy.

**Supplementary Information:**

The online version contains supplementary material available at 10.1007/s00268-021-06372-1.

## Introduction

Currently, minimally invasive techniques are commonly used in gastrointestinal surgery and have also become increasingly popular in pancreatic surgery, even in pancreatoduodenectomy (PD), the most complex procedure.

In laparoscopic pancreatoduodenectomy, reconstruction is most challenging even for experienced surgeons, because pancreatojejunostomy or pancreatogastrostomy and hepaticojejunostomy have to be performed handsewn intracorporeally, whereas gastrojejunal anastomosis can be achieved laparoscopically by stapler. Laparoscopic pancreaticoduodenectomy is therefore associated with an increased rate of complications due to pancreatic fistula [1], and the learning curve for this complex procedure is yet to be defined [2].

Minimally invasive hybrid techniques as in two field esophagectomy, where a laparoscopic abdominal resection phase is combined with an open reconstruction via thoracotomy, have shown advantages in respect of pulmonary complications and global health owing to the reduced surgical trauma of laparoscopy [3] on the one hand and safe and easy implementation on the other hand.

Hybrid laparoscopic pancreatoduodenectomy is used to combine the advantages of laparoscopic pancreatoduodenal resection in combination with a well established open and safe reconstruction through a midline mini-laparotomy. Via this midline mini-laparotomy the specimen is removed, and the technically very demanding laparoscopic reconstruction is avoided [4].

The aim of this meta-analysis was to compare the results of hybrid laparoscopic pancreatoduodenectomy (HPD) versus open pancreatoduodenectomy (OPD) in comparative studies as well as to evaluate the results of hybrid laparoscopic pancreatoduodenectomy in published series.

## Methods

### Operation

Hybrid laparoscopic pancreatoduodenectomy is defined as a surgical technique in which the dissection phase is performed laparoscopically. The open reconstructions were performed via midline mini-laparotomies, varying in length between 4 and 10 cm, in the same way as in open surgery. Different techniques of anastomoses (hepaticojejunostomy, pancreatogastrostomy or pancreatojejunostomy, duodenojejunostomy or gastrojejunostomy) were used, depending on the preference of the individual institution. The technique of hybrid laparoscopic pancreatoduodenectomy was performed as previously described [5, 6]. Open pancreatoduodenectomy was performed according to the preference of the institutions as pylorus-preserving pancreatoduodenectomy or subtotal stomach preserving pancreatoduodenectomy, and also here different anastomotic techniques were used. Neither type nor length of the incision was specified in the studies, with one exception, where a long upper midline incision or an inverted-L incision were described.

Indications for hybrid PD were benign as well as malignant pathologies of the pancreatic head (Supplemental Table 1). Selection criteria for hybrid laparoscopic PD were defined by the operating institutions. Mainly patients with small lesions of the pancreatic head without perivascular invasion and who did not have previous extensive upper abdominal surgery were referred for hybrid laparoscopic PD.

### Literature search

A systematic literature research was performed according to PRISMA guidelines [7] in the PubMed database for studies published until March 1, 2021. Search terms used were “laparoscopy” (approach) and “pancreatoduodenectomy” (procedure). Titles, abstracts and text of the articles were screened based on inclusion and exclusion criteria. Only studies comparing hybrid pancreatoduodenectomy with open pancreatoduodenectomy, case series and case reports, were included. If repeatedly published series were found, only the most recent study was considered. Also articles after manual search in reference lists of related meta-analyses [8–11] and systematic review articles were included. Articles in other languages than English, duplicate articles, not relevant articles, studies on animals were excluded. Comparative studies were only included if at least one outcome variable of interest was reported.

### Assessment of quality of studies

Only non-randomized studies were detected. The quality of the studies was appraised according to Cochrane guidelines, and the risk of bias has been classified in high, unclear, or low risk of bias [12]. Additionally, all comparative studies were assessed according to the Maastricht-Amsterdam criteria. Included studies were ranked with a maximum of 19 points. Studies with a score less than 9 were considered of low quality.

### Data extraction

Extracted outcome data of interest in this meta-analysis included operative outcomes (operative time, intraoperative blood loss, intraoperative transfusion, conversion to open approach) and postoperative outcomes (mortality, pancreatic fistula B/C [13], postpancreatectomy hemorrhage [14], delayed gastric emptying [15], hepaticoenterostomy leakage, overall complications, reoperation, morbidity classified according Clavien–Dindo Grade 2 to 5 [16], surgical site infections, length of hospital stay).

### Statistical analysis

Meta-analysis for comparative studies was performed according to recommendations of Cochrane guidelines and statistically processed by the metafor package [17] for R software.

Categorical data were presented as frequencies and percentages. Continuous data were presented as stated in included original articles.

Mean and standard deviation were extracted from a study when available. *I*^2^ was used to quantify heterogeneity between studies (Tables 1 and 2) [18]. For low and moderate heterogeneity (*I*^2^<50 %), the fixed effect model was used. In case of considerable heterogeneity (*I*^2^>50 %), the random effect model was used. In forest plots, estimates were expressed as weighted mean difference for continuous data and odds ratio for event related outcomes and were all reported with corresponding 95 % confidence intervals (CI).

**Table 1:** Heterogeneity analysis of all included studies

Parameter	*I*^2^ value	*p* value, *I*^2^ test
Mortality	0	0,998
DGE	61,509	0,001
POPF B/C	34,07	0,14
PPH	0	0,857
Transfusion	50,999	0,005
Hepatic leak	0	0,855
Reoperation	0	0,861
SSI	89,022	0
Clavien–Dindo	38,697	0,081
Operative time	99,13	0
Complications	88,297	0
Conversion	93,764	0
Blood loss	93,543	0
OHS	0	0,682

*I*^2^ value heterogeneity measure; *p* value of *I*^2^ test (*p* < 0,05 significant)

**Table 2:** Heterogeneity analysis of comparative studies

Parameter	I^2^ value	*p* value, *I*^2^ test	OR.FE.p	OR.RE.p
Mortality	0	0,864	0,961	0,961
DGE	14,358	0,323	0,415	0,553
POPF B/C	32,181	0,182	0,261	0,415
PPH	0	0,963	0,397	0,397
Transfusion	20,348	0,28	0,211	0,211
Hepatic leak	1,453	0,362	0,891	0,891
Reoperation	0	0,914	0,563	0,563
SSI	0,779	0,388	0,019	0,019
Clavien–Dindo	0	0,503	0,248	0,248
Operative time	0	0,379	0,013	0,013
Complications	64,555	0,037	0,001	0,096
Conversion	0	0,621	0	0
Blood loss	94,881	0	0	0,097
OHS	0	1	0,429	0,429

*I*^2^ value heterogeneity measure; *p* value of *I*^2^ test (*p* < 0,05 significance); OR.FE.p pvalue in Fixed Effect Model; OR.RE. p *p* value in Random Effect Model

Sensitivity analysis was performed excluding studies of low quality: only studies with at least nine points according to the Amsterdam criteria were included.

## Results

**Fig. 1:**
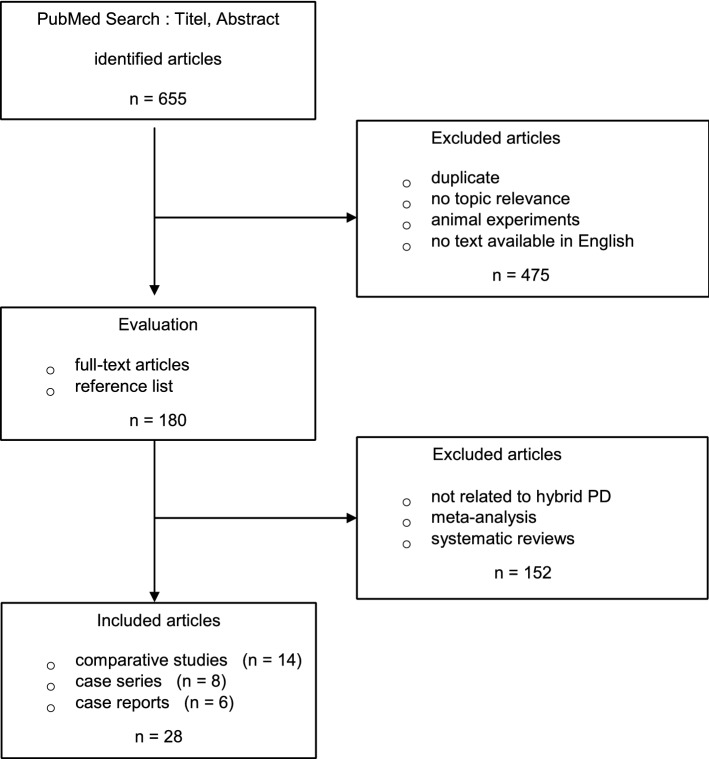
The PRISMA flowchart of literature review

The PRISMA flow diagram is shown in Fig. 1. In total, 655 articles were identified. After screening titles and abstracts 180 articles remained. After evaluation of the full text of these articles and reference lists of meta-analyses and reviews another 152 articles were excluded. Fourteen comparative studies, eight case series, and six case reports with a total of 505 patients were included in the study. Fourteen comparative studies with 371 patients were suitable for meta-analysis.

Descriptive meta-analysis was performed including all available cases of hybrid-laparoscopic pancreatoduodenectomy, also from non-comparative series and reports. Given the relative paucity of comparative data, this enabled to include a larger number of patients.

Characteristics of included studies are shown in Table 3. Quality of studies is shown detailed in supplementary material as Supplemental Tables 2 and 3. Results of heterogeneity for all studies are shown in Tables 1 and 2. Operative and postoperative outcomes for all hybrid pancreatoduodenectomies as well as for comparative studies are shown in the forest plots (Figs. 2, 3, 4, 5, 6, 7, 8, 9, 10, 11, 12 as well as in supplementary materials Suplemental Figures 1–16).

**Table 3:** Overview of all hybrid pancreatoduodenectomies (study characteristics)

Author, Year	*n*	Type of article	Resection		Reconstruction				
			Lap	HA	PG	PJ	HJ	GJ	DJ
Gagner [19], 1994	1	FB	+			Lap	Lap		o
Cuschieri [20], 1994	2	FB	+		?	?	?	?	?
Ammori [21], 2004	1	FB		+	O?	O?	o	O?	O?
Kimura [22], 2005	1	FB		+		o/HA	o/HA		o
Staudacher [23], 2005	4	S	+			o	o		o
Mabrut [24], 2005	3	S	+ 2	+ 1	O?	O?	o	O?	O?
Dulucq [25], 2006	9	KS(TL/LA)	+			o	o	o	
Pugliese [26], 2008	7	KS(TL/LA)	+			o	o	o	o
Cho [27], 2009	15	KS	+			o	o		o
Suzuki [28], 2012	6	S		+	o 3	o 3	o	o	
Asbun [29], 2012	3	KS		+	HA?	HA?	HA?	HA?	HA?
Kuroki [30], 2012	20	KS	+			o	o	o	o
Nakamura [31], 2012	12	S	+			HA	Lap		HA?
Lee [32], 2013	42	S	+		o		Lap		o
Langan [33], 2014	28	KS		+	O?	O?	o		o
Speicher [34], 2014	31?	KS(TL/LA)	+		O?	O?	o	O?	O?
Wang [35], 2014	13	KS	+			o	o	o	
Dokmak [1], 2015	46	KS	+			Lap	Lap	Lap/o	
Liu [36], 2015	21	S	+		o		Lap		o
Mendoza [37], 2015	18	KS	+			o	o	o 2	o 16
Zimmitti [38], 2016	1	FB	+		o		Lap	o	
Koh [39], 2016	1	FB	+			O	o	o	
Patel [40], 2017	17	KS(TL/LA)	+			O	o	o	
Hilst [41], 2018	56	KS(TL/LA)	+			O	o	O?	O?
Deichmann [42], 2018	60	KS	+		o		o		o
Pham [43], 2020	18	S	+			O	o	o	
Wang [44], 2020	48	KS(TL/LA)	+			O	o	o	
Al-Sadairi [45], 2021	21	S	+			O	o	o	

Particularities: Kimura additionally Braun Anastomosis, Liu additionally Braun Anastomosis open

FB case report; S case series; KS comparative study; Lap laparoscopic; HA handassisted; TL total laparoscopic; LA laparoscopic assisted; o open; PG Pancreatogastrostomy; PJ Pancreatojejunostomy; HJ Hepaticojejunostomy; DJ Duodenojejunostomy; GJ Gastrojejunostomy

O? case number of open performed reconstruction not spicified; ? not specified; n case number

**Fig. 2:**
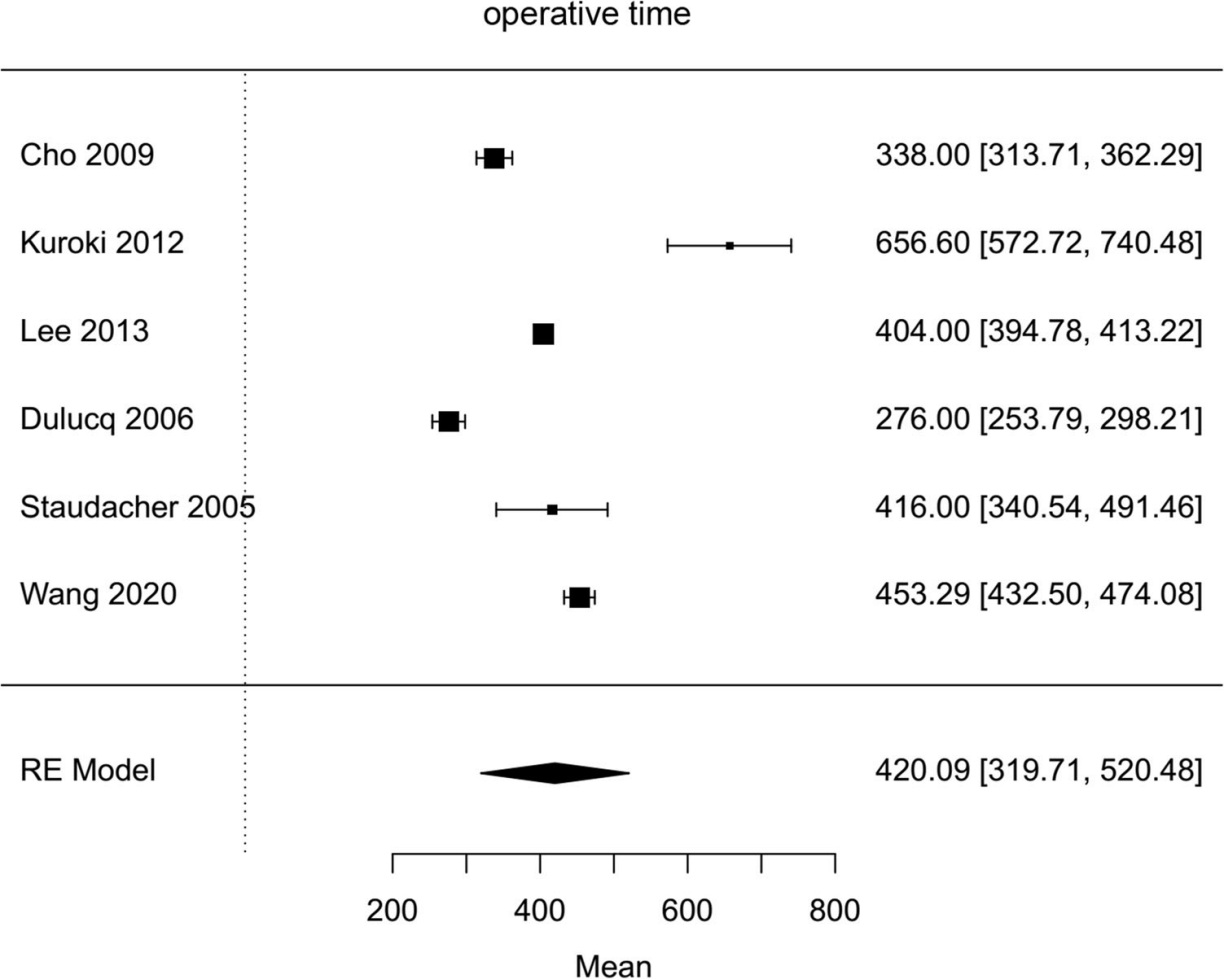
Forest plot; average operative time of all HPD`s in minutes

**Fig. 3:**
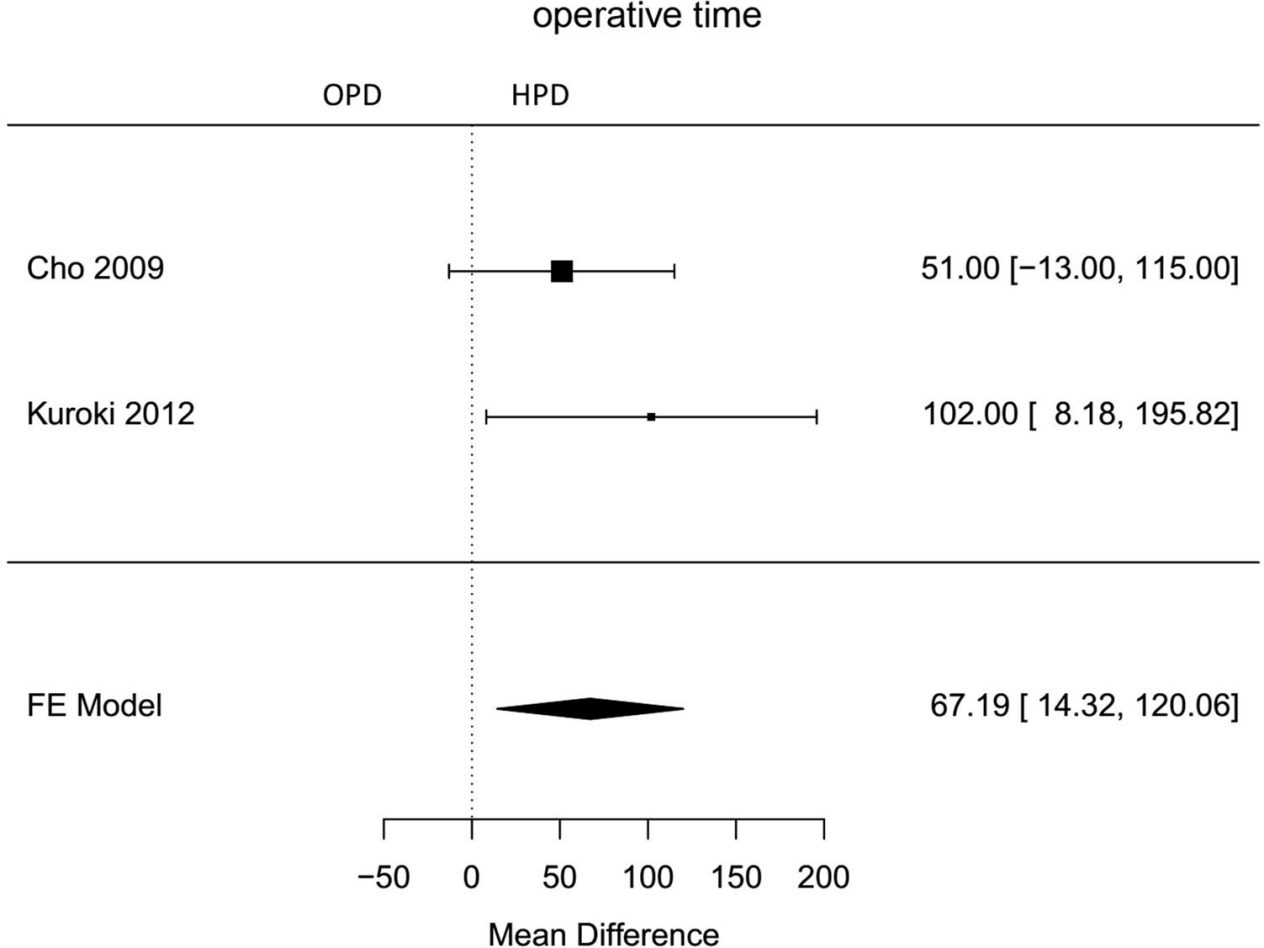
Forest plot; mean difference of operative time in comparative studies (comparison between HPD and OPD)

**Fig. 4:**
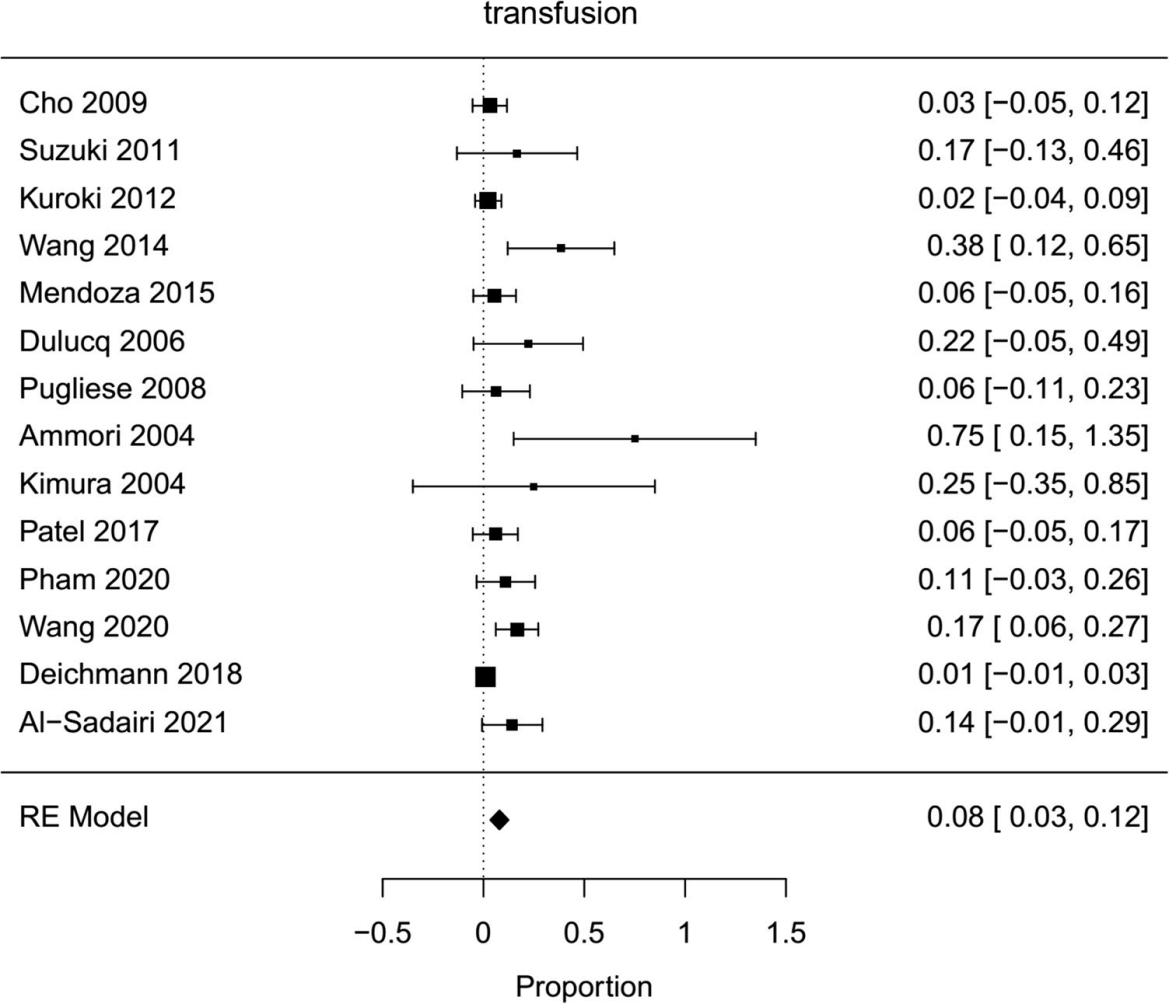
Forest plot; transfusion rate of all HPD`s

**Fig. 5:**
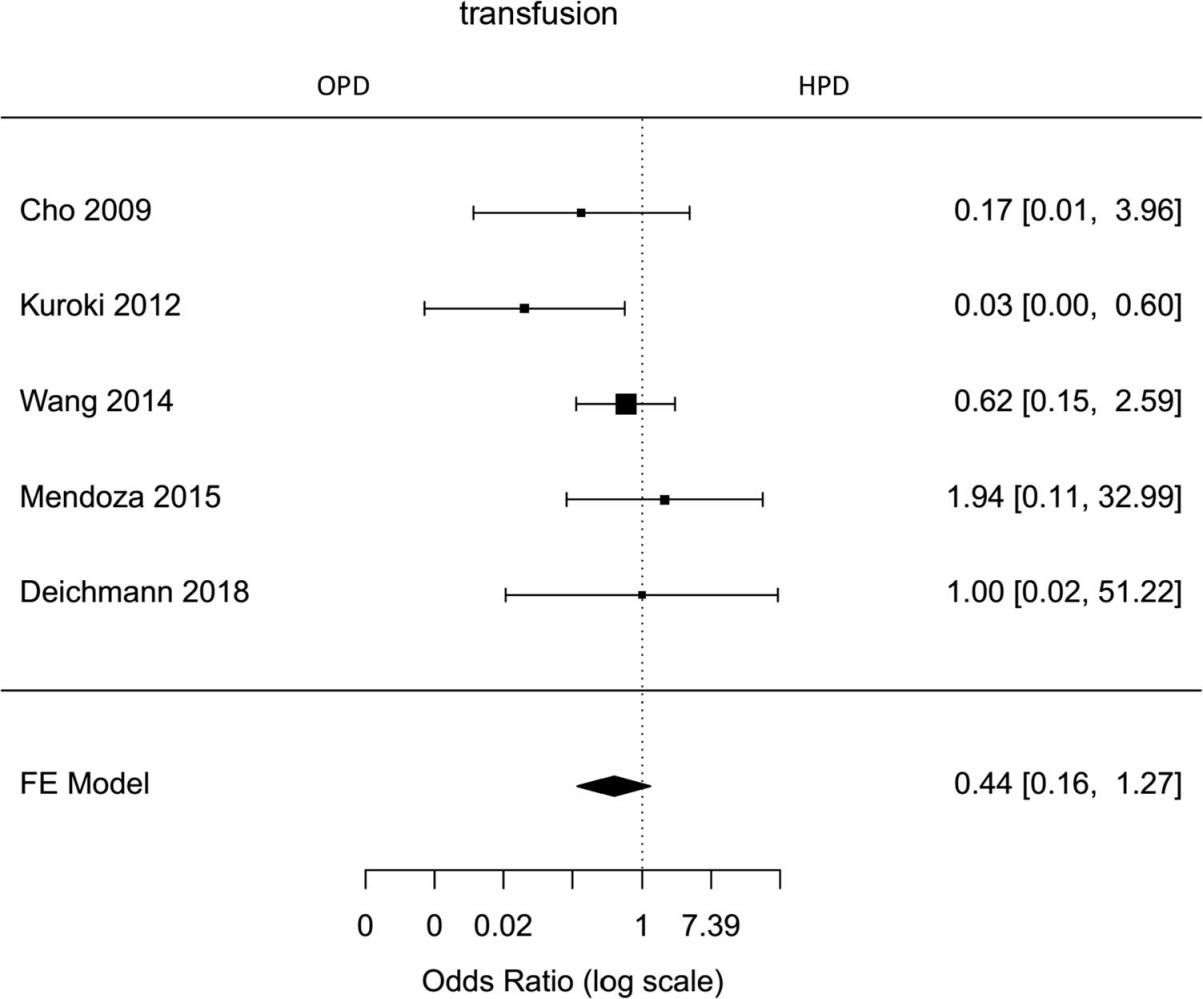
Forest plot; transfusion rate in comparative studies (comparison between HPD and OPD)

**Fig. 6:**
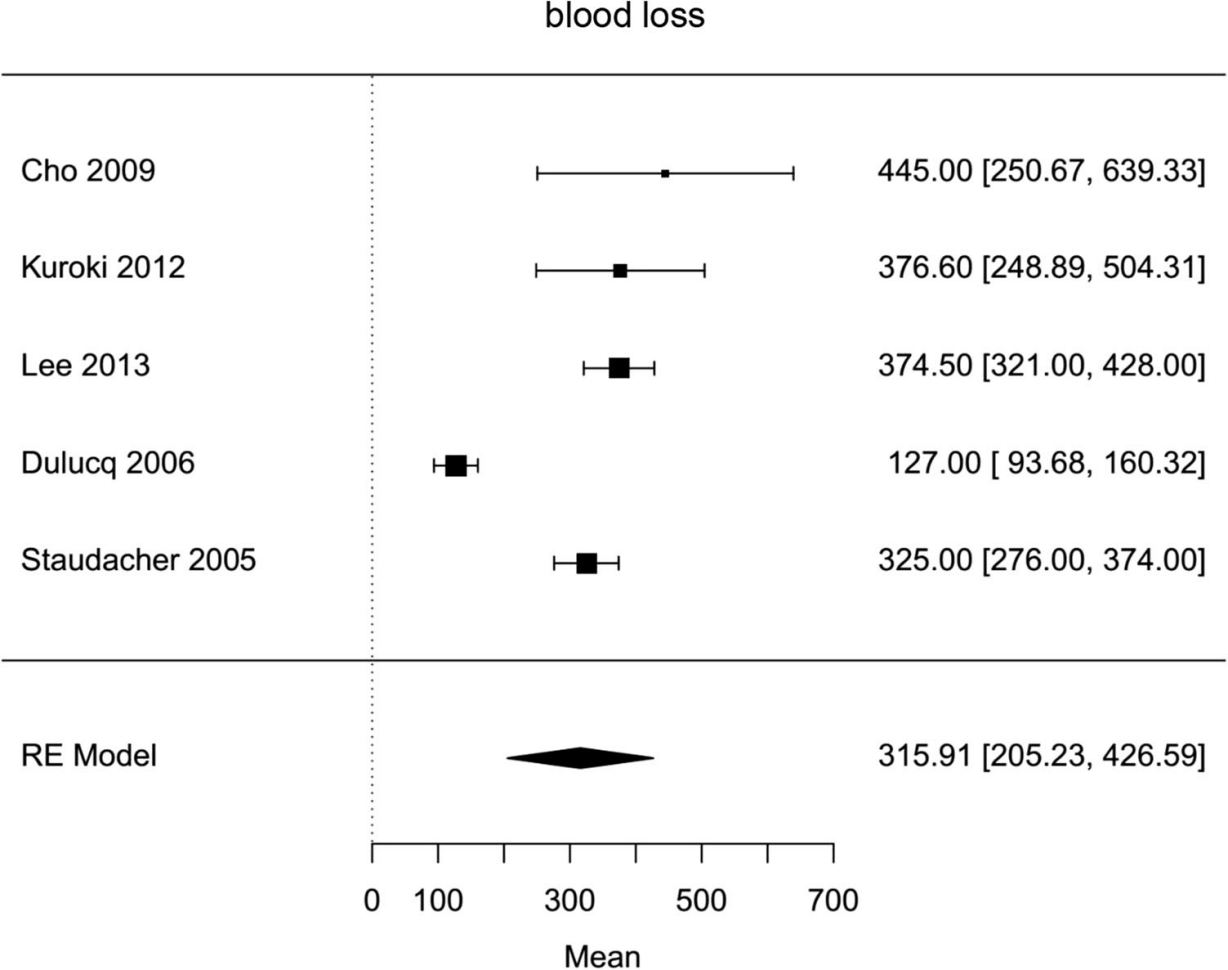
Forest plot; average intraoperative blood loss of all HPD’s in milliliter

**Fig. 7:**
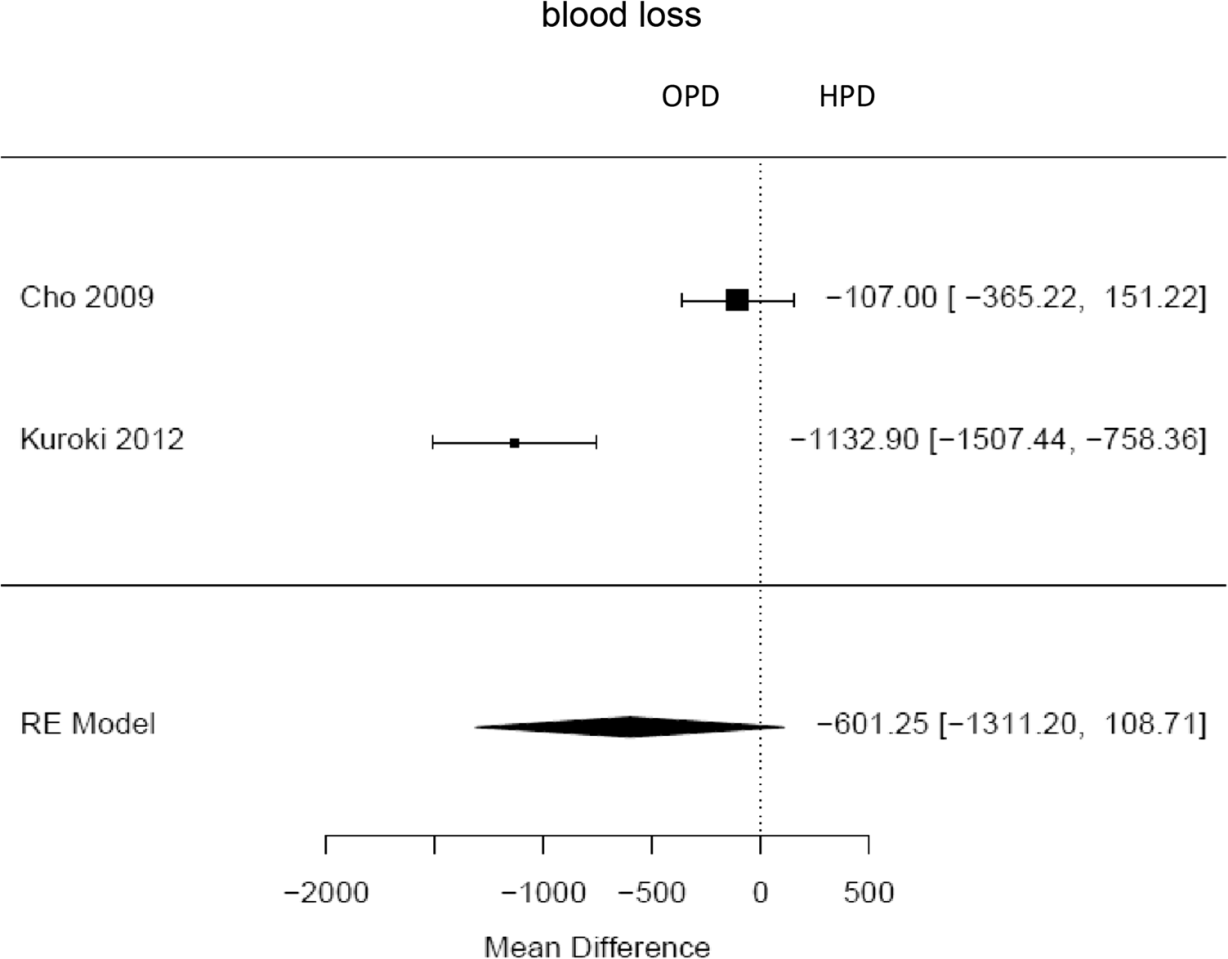
Forest plot; mean difference in intraoperative blood loss between HPD and OPD in milliliter

**Fig. 8:**
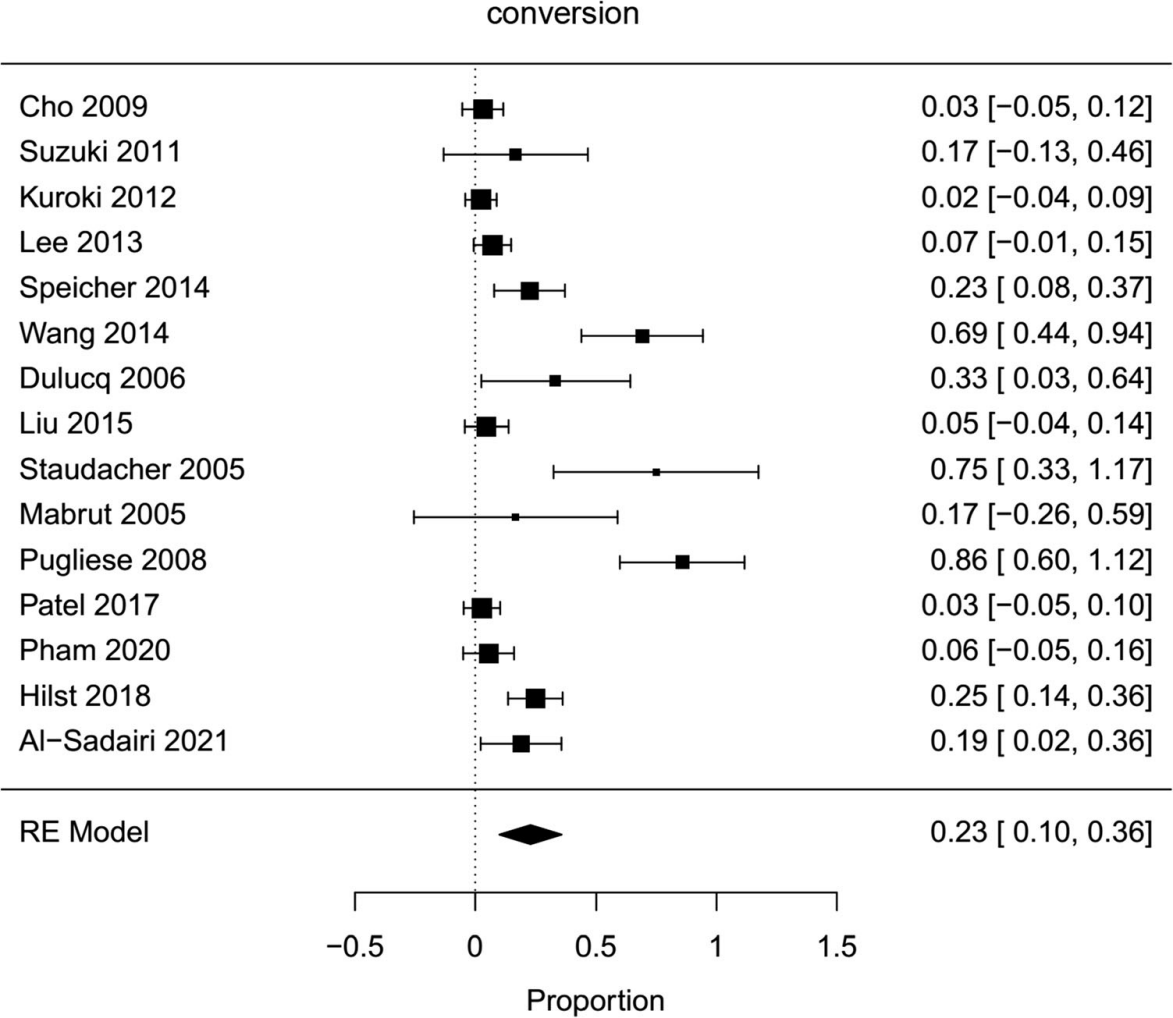
Forest plot; conversion rate of all HPD’s

**Fig. 9:**
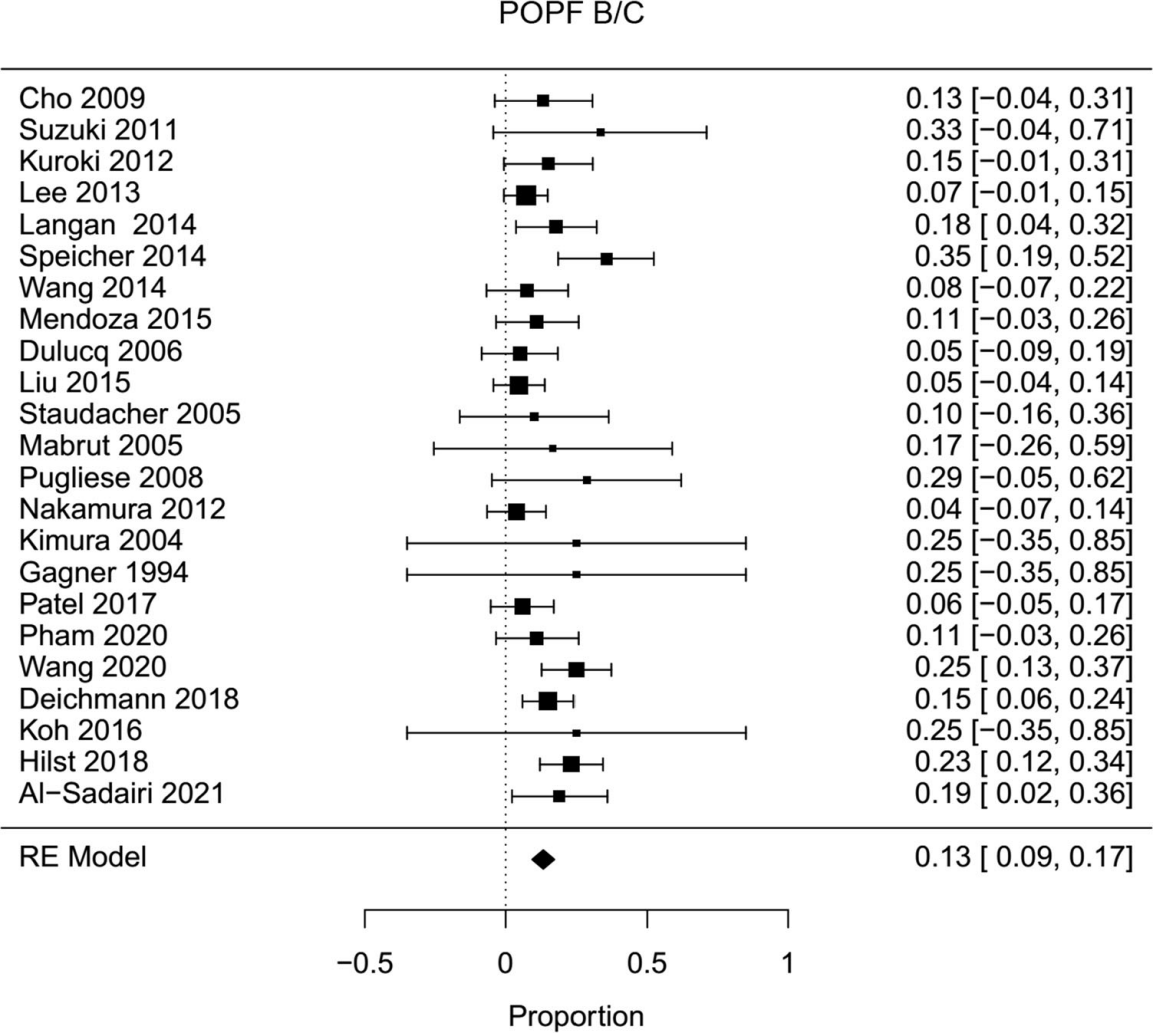
Forest plot; pancreatic fistula B/C rate of all HPD’s

**Fig. 10:**
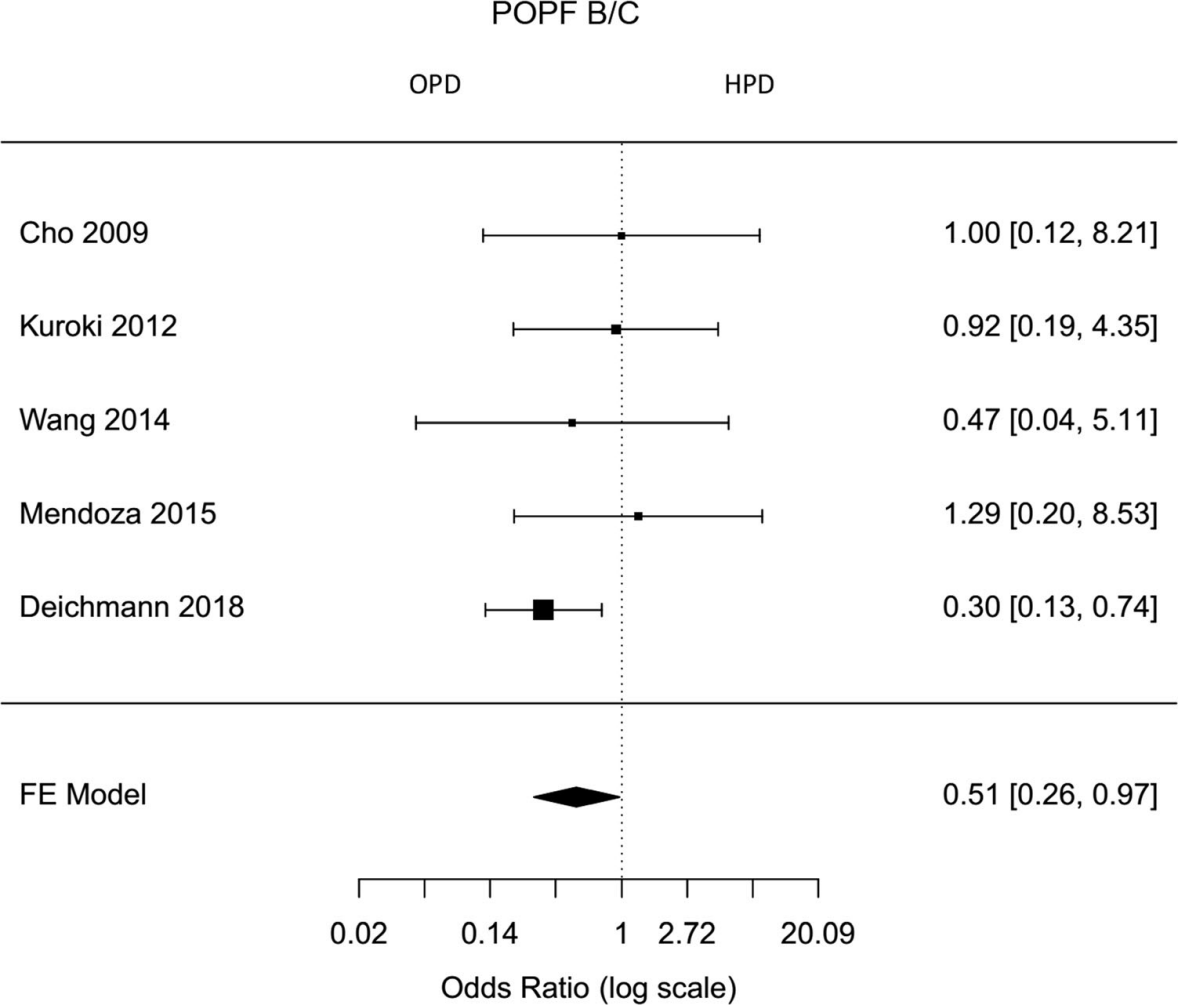
Forest plot; pancreatic fistula B/C rate in comparative studies (comparison between HPD and OPD)

**Fig. 11:**
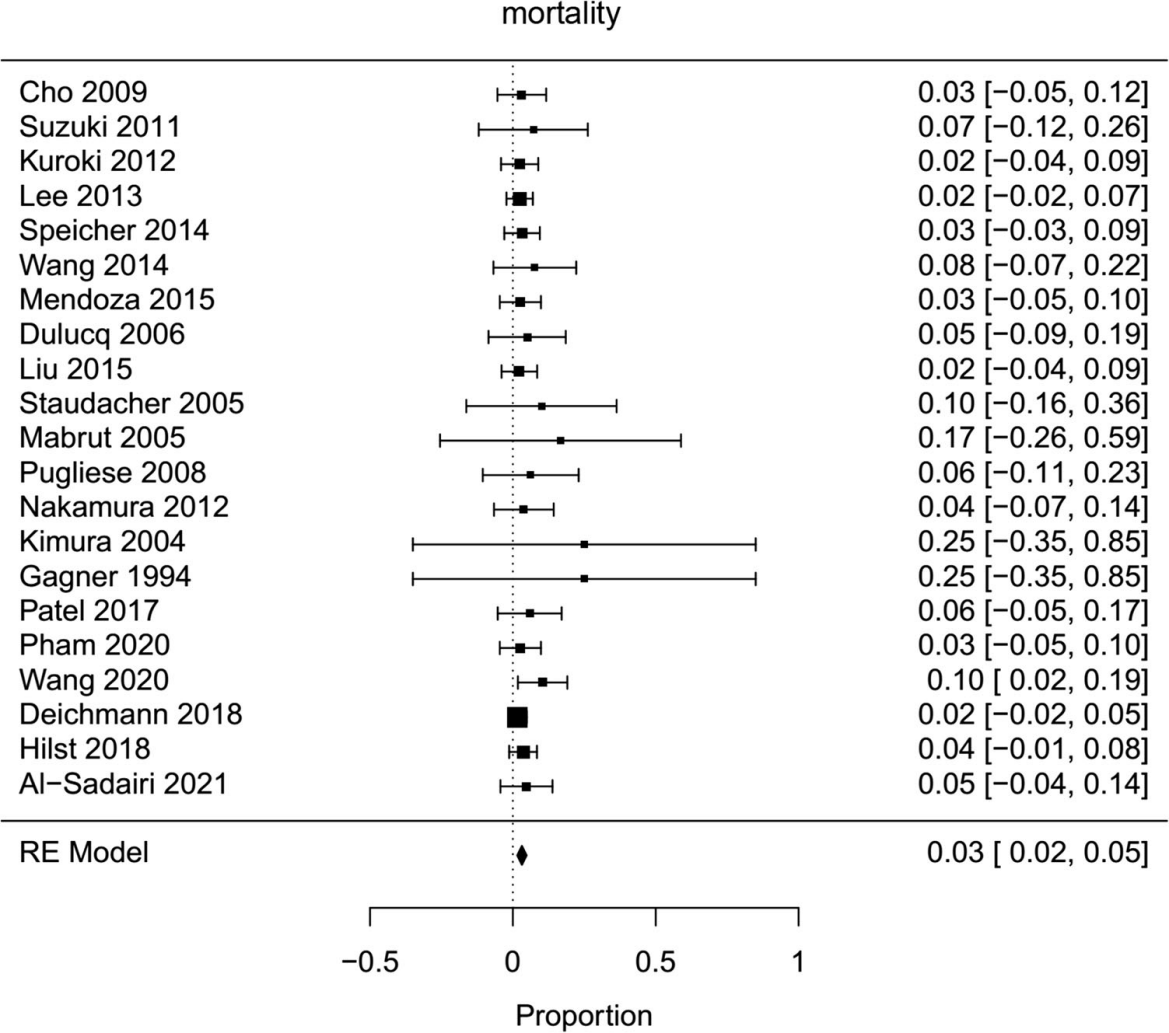
Forest plot; mortality of all HPD’s

**Fig. 12:**
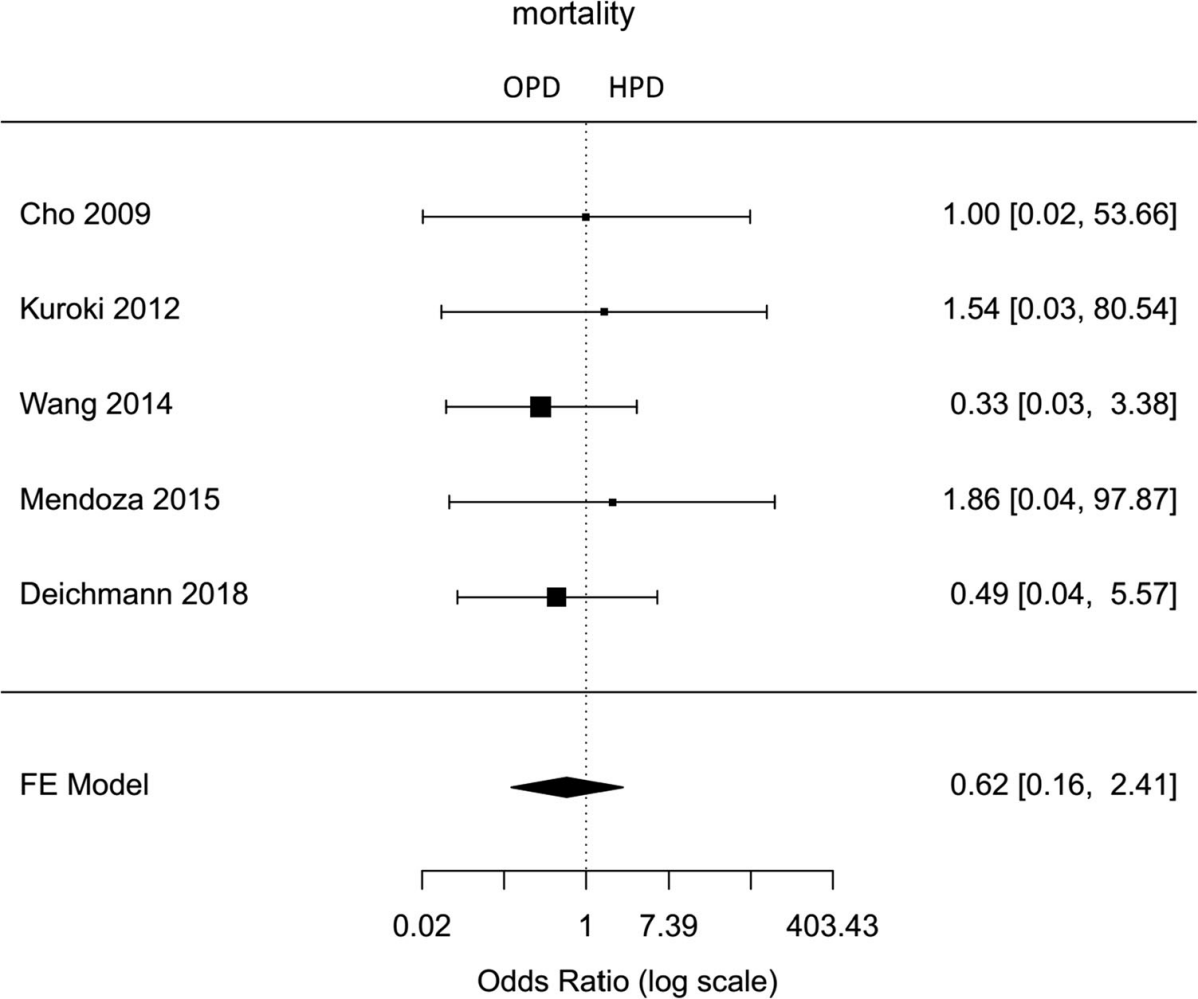
Forest plot; mortality rate in comparative studies (comparison between HPD and OPD)

Most studies were designed as feasibility studies, and long-term outcome was not reported.

The largest matched-pair analysis comparing hybrid PD versus OPD assessing long-term overall survival and oncologic outcome which was presented, found a trend of improved overall survival in patients receiving hybrid PD [42]. Radical oncologic resection could also be performed safely by hybrid PD in cancer patients.

### Operative outcomes

The average operative time estimated for all included hybrid laparoscopic PD was 420 min (95 % CI 319–520). Operative time was significantly longer for hybrid laparoscopic PD compared to open PD (Mean HPD 494,6 min, Mean OPD 421,6 min, mean difference 67 min, 95 % CI 14–120, *p*=0,013). The estimated transfusion rate for all hybrid laparoscopic PD was 8 % (95 % CI 3–12 %). The intraoperative transfusion rate was not significantly lower for hybrid PD (proportion HPD 2%, proportion OPD 1,6 %, OR 0,44, 95 % CI 0,16–1,27, *p*=0,2). Blood loss in HPD was lower, but not significantly (Mean HPD 397,2 ml, Mean OPD 1017,8 ml, MD − 601 ml, 95 % CI − 1311–108, *p*=0,1). For all HPD the conversion rate was estimated with 23 % (95 % CI 10–36 %).

### Postoperative outcomes

In descriptive meta-analysis, complication rates were as follows: pancreatic fistula rate 13 % (Fig. 9), postpancreatectomy hemorrhage 6 % (Supplemental Figure 1), delayed gastric emptying 11 % (Supplemental Figure 5), morbidity (Clavien–Dindo 2-5) 30 % (Supplemental Figure 6), overall complications 47 % (Supplemental Figure 4), mortality 3 % (Fig. 11), and surgical site infection 12 % (Supplemental Figure 7).

The rates of pancreatic fistula, postpancreatectomy hemorrhage, delayed gastric emptying, morbidity (Clavien–Dindo 2–5), overall complications, mortality, and surgical site infection were lower for hybrid laparoscopic PD, but did not differ significantly to open PD (Fig. 10, Supplemental Figures 2, 9, 10, 8, Fig. 12, Supplemental Figure 3).

For all hybrid laparoscopic PD, the rate of hepaticoenterostomy leakage was 3 % (Supplemental Figure 11) and the rate for reoperation was 7 % (Supplemental Figure 15). Both rates were higher in hybrid PD, but also differed not significantly from open PD in comparative studies (Supplemental Figures 12 and 16).

Mean overall hospital stay for all hybrid PD was 16,68 days (Supplemental Figure 13). The only evaluable comparative study showed, that overall hospital stay was longer in the hybrid PD group without statistically significance (Supplemental Figure 14).

## Discussion

Laparoscopic gastrointestinal surgery has been demonstrated to result in reduced postoperative pain, shorter hospital stay, rapid return to baseline performance status, and reduced morbidity with oncological equivalency, when compared to the traditional open procedure. Laparoscopic procedures, therefore, have rapidly gained widespread acceptance [46].

The use of minimally invasive pancreatoduodenectomy (MIPD) has increased but is still performed by a limited number of institutions and is lagging compared with the implementation of laparoscopic distal pancreratectomy.

Laparoscopic pancreatoduodenectomy is very challenging even for experienced pancreatic surgeons because three difficult laparoscopic anastomoses must be performed with potentially life-threatening complications [47].

The learning curve for this complex procedure is not yet defined [2]. In centers performing less than 10 MIPDs annually, this technique was associated with higher perioperative mortality compared with open pancreatoduodenectomy [40, 41]. Publications of series of more than 20 cases demonstrate a decrease in the average operative time, showing that pancreatic surgeons with minimally invasive expertise may be able to perform MIPD with similar operative times as open procedures [48]. A retrospective multicenter propensity matched cohort study comparing MIPD cases to OPD controls from European centers, performing at least 10 MIPDs per year, found no differences in major morbidity, mortality and length of stay between MIPD and OPD. MIPD was, however, associated with a 10 % higher rate of grade B/C POPF and longer operative times.

In a previous analysis there were no differences in outcomes between laparoscopic-assisted, robot-assisted-, and hybrid-PD (major morbidity: 27 % vs. 27 % vs. 35 %; POPF: 24 % vs. 19 % vs. 25 %; 30-day mortality: 2,9 % vs. 5,2 % vs. 5,4 %). Single-row pancreatojejunostomy was identified as a risk factor for POPF in MIPD [47]. One published randomized trial on laparoscopic versus OPD for periampullary tumors was underpowered to demonstrate the benefit regarding major morbidity [49]. Improved outcomes in centers performing more than 40 OPDs annually could indicate that the optimal minimum quantity for MIPD is also higher [47].

A cutoff of 20 MIPD was recommended in an international survey among 435 pancreatic surgeons [50], and a minimum of 20 totally MIPD was also decided to be the cutoff to participate in the LEOPARD-2 trial [51]. This first randomized controlled multicenter study was designed to assess whether MIPD reduces time to functional recovery as compared with OPD. The study protocol allowed laparoscopic surgery and robot-assisted surgery because both were considered equivalent methods of MIPD [52]. The LEOPARD-2 trial was prematurely terminated because laparoscopic pancreatoduodenectomy was associated with more complication-related deaths than open pancreatoduodenectomy [51]. Experience, learning curve, and volume influenced the outcome.

All of these studies well demonstrate that even in well-established training settings the learning curve is flat and a high number of cases are needed to implement safe and reproducible results.

To improve the results of MIPD and disseminate MIPD, application of dedicated programs might be useful as has been previously shown in the Netherlands for laparoscopic distal pancreatectomy (LAELAPS) [53]. A volume–outcome relation seems to be stronger for laparoscopic pancreatoduodenectomy, so that the LEOPARD-2 trial participating centers were required to take part in a training programme (LAELAPS-2) and performed a median of 19 (range 15–23) laparoscopic pancreatoduodenectomies annually [54]. During the trial, the randomization decreased this annual volume per participating center to a median of 11 (range 6–15) procedures. Therefore a negative influence of this reduction in center volume cannot be ruled out [51].

In addition, hybrid laparoscopic pancreatoduodenectomy is used in the implementation phase of minimally invasive PD to combine the potential advantages of laparoscopic pancreatoduodenal resection with well-established open and safe reconstruction. The hybrid approach aims to avoid the technically demanding laparoscopic anastomoses. Because there are no prospective randomized controlled studies comparing the open approach to the hybrid laparoscopic technique, this meta-analysis was performed as the best means to gather more evidence.

The presented systematic review of the literature revealed 14 comparative studies: eight case series and six case reports. The definition of a high-volume center of pancreatic surgery varies widely and various cutoffs for defining high-volume centers are used. The best model of high-volume centers was an annual institution resection volume of 19 or more. Based on the information provided within the included comparative studies, all institutions crossed a cutoff of 20 pancreatic resections annually. So all institutions met the criterion of a high volume center. Due to the application of metafor package in R [17] for statistical analysis, the detected studies could be included in the meta-analysis. Therefore, included studies are heterogeneous and selection bias was a common problem. According to Cochrane guidelines and Maastrich-Amsterdam criteria, the included studies were rated with moderate quality.

For comparison of operative time, only two studies specifying mean operative time were available. These studies showed a significantly longer operative time for hybrid laparoscopic pancreatoduodenectomy. Due to the complexity of the intervention, this took approximately 1 h longer than the open procedure. As mentioned above, unlike many other laparoscopic procedures, laparoscopic pancreatoduodenectomy seems to require a very long learning period [34]. The largest matched-pair analysis comparing hybrid versus open pancreatoduodenectomy was the first demonstrating a significantly shorter median operative time for the hybrid pancreatoduodenectomy 352 min versus 397 min for open pancreatoduodenectomy [42]. This is supported by the lowest mortality rate in studies with higher case numbers [30, 32, 42]. The calculated mortality rate in this meta-analysis was not statisticaly significant higher for open approach with Odds ratio of 0,62 (proportion HPD 2%, proportion OPD 2%, 95 % CI 0,16–2,41). According to data of the National Cancer Database of the USA, mortality of laparoscopic pancreatoduodenectomy was 4,8 % compared to 3,7 % after open pancreatoduodenectomy [55]. The higher mortality rate occurred in hospitals with less than 10 laparoscopic pancreatic resections per year [56], again underlining the complex learning curve of this procedure.

Hybrid laparoscopic pancreatoduodenectomy showed lower transfusion rate and blood loss, which were not statistically significant. This can be explained by magnification of the visible field of laparoscopy and has been shown for other laparoscopic approaches in similar fashion.

In this meta-analysis, the estimated overall conversion rate from hybrid laparoscopic to open pancreatoduodenectomy was still high with 23 % and may be interpreted in part as a result of patient selection. Frequently, pancreatic adhesions to the mesentericoportal vein resulted in preemptive conversion to the open approach [57]; however, the decisions for conversion were not mentioned in every study reported.

Postoperative outcomes were comparable for both techniques. In meta-analysis of comparative studies, the insufficiency rate of pancreatic anastomosis—a potentially life-threatening complication—was lower but not significantly lower after minimally invasive pancreatoduodenectomy (proportion HPD 16 %, proportion OPD 20 %, Odds ratio 0,51, 95 % CI 0,26–0,97). The leakage rate for hepaticoenterostomy after the hybrid approach was higher, but again not statistically significant (proportion HPD 2 %, proportion OPD 2 %, Odds ratio 1,14, 95 % CI 0,18–7,07). This is not unexpected, because in both operative techniques open pancreatogastrostomy or pancreatojejunostomy were performed. Estimated risk of pancreatic fistula in all analyzed articles differed between 4 and 35 % [31, 34]. Possible reasons may be different anastomotic techniques. An insufficiency of the pancreatic anastomosis with following abscess or bleeding are the main reasons for reoperations. Results of this meta-analysis showed for all hybrid laparoscopic pancreatoduodenectomy a reoperation rate of 7 %. In comparative studies, reoperation rate after laparoscopic and open pancreatoduodenectomy were comparable for both groups (proportion HPD 4 %, proportion OPD 4 %, Odds ratio 1,24, 95 % CI 0,07–20,57). Postpancreatectomy hemorrhage was lower after a hybrid approach without statistical significance (proportion HPD 4 %, proportion OPD 5 %, Odds ratio 0,64, 95 % CI 0,23–1,80). Clinically relevant delayed gastric emptying occurred less frequently in hybrid pancreatoduodenectomy, but it varied not significantly different from open approach (proportion HPD 7 %, proportion OPD 8 %, Odds ratio 0,71, 95 % CI 0,31–1,63).

Despite a smaller incision used for mini-laparotomy in hybrid technique, surgical site infection rate was not significantly higher in the open technique (proportion HPD 19 %, proportion 13 %, Odds ratio 0,67, 95 % CI 0,11–3,97).

A statistical analysis of length of hospital stay was possible only in one comparative study. Mean hospital stay was 16,4 ± 3,7 days for hybrid pancreatoduodenectomy and 15,6 ± 1,3 days for the open approach. The difference of 0,8 days was not statistically significant. The authors postulate that there will be advantages for the duration of hospital stay for laparoscopy with increasing experience [27].

Although this meta-analysis showed no benefit of hybrid pancreatoduodenectomy, the open reconstruction may provide an alternative for, or a step-up technique to laparoscopic/robot-assisted surgery during the learning curve, because there was no disadvantage concerning safety of the reconstruction even in the reported series that might represent the learning curve of several centers [58]. The next step should be a comparison of laparoscopic versus robotic PD. Although the frequency of robotic operations increases, it is not feasiable at the moment, because comparative studies are lacking. Experience and operative frequency influenced the outcome and improved the results of MIPD. The largest mached pair analyses comparing hybrid pancreatoduodenectomy with open pancreatoduodenectomy in 120 patients showed a reduction in clinically relevant postoperative complications, faster recovery for the hybrid technique and an equal long-term onologic outcome [42]. Therefore, a hybrid pancreatoduodenectomy can be considered as a save transitional procedure to total laparoscopic or robot-assisted pancreatoduodenectomy. This further development should be investigated in a prospective study. Their implantation is likely to be difficult, as the data of the interrupted LEOPARD-2 Study showed.

## Conclusions

Data determined in this meta-analysis advised the implementation of hybrid pancreatoduodenectomy, although this study has restrictions due to missing prospective studies. This meta-analysis demonstrates significantly increased operation time for hybrid laparoscopic pancreatoduodenectomy, while major morbidity remains comparable to open technique. Overall results of this meta-analysis demonstrated the hybrid technique as a safe procedure in high-volume centers with adequate numbers of operations offering aspects of a safe transition to fully laparoscopic/robotic pancreatoduodenectomy.

## Supplementary Information

Below is the link to the electronic supplementary material.

Supplementary file1 (DOCX 2068 kb)
