# Supplementary material for: Hybrid Laparoscopic Versus Open Pancreatoduodenectomy. A Meta-Analysis
Source: World J Surg. 2022 Jan 18;46(4):901–15. doi: 10.1007/s00268-021-06372-1 (PMC8885482; doi:10.1007/s00268-021-06372-1)
Supplement: Supplementary file 1 — Supplementary file1 (DOCX 2068 kb) [file 268_2021_6372_MOESM1_ESM.docx]

Supplementary Materials

Supplemental Table 1: Pathologic results in comparative Studies

| Author, Year | n | Type of article | Pathology | | | | | | | | | | | | | |
| --- | --- | --- | --- | --- | --- | --- | --- | --- | --- | --- | --- | --- | --- | --- | --- | --- |
|  |  |  | Ductal adenocarcinoma | | Periampullary carcinoma | | cholangiocarcinoma | | Cystic neoplasia | | Cnronic pancreatitis | | Neuroendocrine tumor | | other | |
|  |  |  | HPD | OPD | HPD | OPD | HPD | OPD | HPD | OPD | HPD | OPD | HPD | OPD | HPD | OPD |
|  |  |  |  |  |  |  |  |  |  |  |  |  |  |  |  |  |
|  |  |  | n | n | n | n | n | n | n | n | n | n | n | n | n | n |
| Cho [27], 2009 | 15 | KS | 1 | 2 | 1 | 5 | 0 | 4 | 9 | 4 | 0 | 0 | 2 | 0 | 2 | 0 |
| Asbun [29], 2012 | 3 | KS | 22 | 100 | 8 | 34 | 3 | 9 | 10 | 36 | 2 | 20 | 6 | 11 | 2 | 5 |
| Kuroki [30], 2012 | 20 | KS | 0 | 4 | 5 | 1 | 8 | 18 | 6 | 7 | 0 | 1 | 1 | 0 |  |  |
| Langan [33], 2014 | 28 | KS | 9 | 8 | 6 | 7 | 1 | 1 |  |  |  |  |  |  | 12 | 9 |
| Wang [35], 2014 | 13 | KS | 9 | 11 | 1 | 3 |  |  | 1 | 3 | 1 | 0 | 0 | 1 | 1 | 2 |
| Dokmak [1], 2015 | 46 | KS | 15 | 14 | 12 | 12 | 3 | 5 | 6 | 8 |  |  | 6 | 5 | 4 | 2 |
| Mendoza [37], 2015 | 18 | KS | 12 | 30 |  |  |  |  | 2 | 1 |  |  |  |  | 4 | 3 |
| Deichmann [42], 2018 | 60 | KS | 12 | 13 | 14 | 17 |  |  | 16 | 5 | 4 | 9 | 5 | 6 | 9 | 10 |

KS comparative study; HPD hybrid pancreatoduodenectomy; OPD open pancreatoduodenectomy

Supplemental Table 2: Risk of bias in included comparative studies

|  | Random sequence generation (selection bias) | Alocation concealment (selection bias) | Blinding of participan  ts and personnel (performance bias | Blinding of ouctome assessment (detection bias) | Incomplete outcame data (attrition bias) | Selective reporting (reporting bias) | Other causes for bias |
| --- | --- | --- | --- | --- | --- | --- | --- |
| Author, Year |  |  |  |  |  |  |  |
| Dulucq, 2006 | x | x | x | x |  |  | ? |
| Pugliese, 2008 | x | x | x | x |  |  | ? |
| Cho, 2009 | x | x | x | x |  |  | ? |
| Asbun, 2012 | x | x | x | x | x | x | x |
| Kuroki, 2012 | x | x | x | x |  |  | ? |
| Langan, 2014 | x | x |  |  |  | x | ? |
| Speicher, 2014 | x | x | x | x |  |  | ? |
| Wang, 2014 | x | x | x | x |  |  | ? |
| Dokmak, 2015 | x | x | x | x |  |  | ? |
| Mendoza, 2015 | x | x | x | x |  |  | ? |
| Patel, 2017 | x | x | x | x |  |  | ? |
| Hilst, 2018 | x | x | x | x |  |  | ? |
| Deichmann, 2018 | x | x | x | x |  |  | ? |
| Wang, 2020 | x | x | x | x |  |  | ? |

x high risk of bias

 low risk of bias

? unclear risk of bias

Supplemental Table 3: Quality of study according to Maastrich-Amsterdam criteria

| KS | Patient selection | | | | Intervention | | | | | Outcome Measurement | | | | | | | Statistics | | | Total score |
| --- | --- | --- | --- | --- | --- | --- | --- | --- | --- | --- | --- | --- | --- | --- | --- | --- | --- | --- | --- | --- |
| M-A Criteria | A | B1 | B2 | C | D | E | F | G | H | I | J | K | L | M1 | M2 | N | O | P | Q |  |
|  |  |  |  |  |  |  |  |  |  |  |  |  |  |  |  |  |  |  |  |  |
| Dulucq, 2006 | 0 | 0 | 0 | 0 | 0 | 0 | 0 | 1 | 0 | 0 | 1 | 0 | 0 | 1 | 1 | 0 | 0 | 0 | 0 | 4 |
| Pugliese, 2008 | 0 | 0 | 0 | 0 | 0 | 0 | 0 | 1 | 0 | 0 | 0 | 0 | 0 | 1 | 0 | 0 | 0 | 0 | 0 | 2 |
| Cho, 2009 | 1 | 0 | 0 | 1 | 1 | 0 | 0 | 1 | 0 | 0 | 1 | 0 | 1 | 1 | 0 | 1 | 1 | 0 | 0 | 9 |
| Kuroki, 2012 | 1 | 0 | 0 | 1 | 1 | 0 | 0 | 1 | 0 | 0 | 1 | 0 | 1 | 1 | 0 | 1 | 1 | 0 | 0 | 9 |
| Langan, 2014 | 1 | 0 | 0 | 1 | 1 | 0 | 0 | 1 | 0 | 1 | 1 | 0 | 1 | 1 | 1 | 1 | 1 | 0 | 0 | 11 |
| Speicher, 2014 | 1 | 0 | 0 | 1 | 1 | 0 | 0 | 1 | 0 | 0 | 1 | 0 | 1 | 1 | 0 | 1 | 1 | 0 | 0 | 9 |
| Wang, 2014 | 1 | 0 | 0 | 0 | 1 | 0 | 0 | 1 | 0 | 0 | 1 | 0 | 1 | 1 | 0 | 1 | 1 | 0 | 0 | 8 |
| Dokmak, 2015 | 1 | 0 | 0 | 1 | 1 | 0 | 0 | 1 | 0 | 0 | 1 | 0 | 1 | 1 | 0 | 1 | 1 | 0 | 0 | 9 |
| Mendoza, 2015 | 1 | 0 | 0 | 1 | 1 | 0 | 0 | 1 | 0 | 0 | 1 | 0 | 1 | 1 | 0 | 1 | 1 | 0 | 0 | 9 |
| Patel, 2017 | 1 | 0 | 0 | 1 | 1 | 0 | 0 | 1 | 0 | 0 | 1 | 0 | 1 | 1 | 1 | 1 | 1 | 0 | 0 | 10 |
| Hilst, 2018 | 1 | 0 | 0 | 1 | 1 | 0 | 0 | 1 | 0 | 0 | 1 | 0 | 1 | 1 | 0 | 1 | 1 | 0 | 0 | 9 |
| Deichmann, 2018 | 1 | 0 | 0 | 1 | 1 | 0 | 0 | 1 | 0 | 0 | 1 | 0 | 1 | 1 | 1 | 1 | 1 | 0 | 0 | 10 |
| Wang, 2020 | 1 | 0 | 0 | 1 | 1 | 0 | 0 | 1 | 0 | 0 | 1 | 0 | 1 | 1 | 1 | 1 | 1 | 0 | 0 | 10 |

KS comparative study; M-A Criteria Maastricht-Amsterdam criteria

Maastricht-Amsterdam criteria list

Patient Selection

A: Were the eligibility criteria specified

B1: Was a method of randomization performed

B2: Was the treatment allocation concealed

C: Were the groups similar at baseline regarding the most important prognostic indicators

Intervention

D: Were the index and control interventions explicitly described

E: Was the care provider blinded to the intervention

F: Were co-interventions avoided or comparable

G: Was the compliance acceptable in all groups

H: Was the patient blinded to intervention

Outcome Measurement

I: Was the outcome assessor blinded to the intervention

J: Were the outcome measures relevant

K: Were adverse effects described

L: Was the withdrawal/drop-out rate described and acceptable

M1: Was a short term follow-up measurement performed

M2: Was a long term follow-up measurement performed

N: Was the timing of the outcome measurement in both groups comparable

Statistics

O: Was the sample size for each group described

P: Did the analysis include an intention-to-treat analysis

Q: Were point estimates and measures of variability presented for the primary outcome measures

# PPH


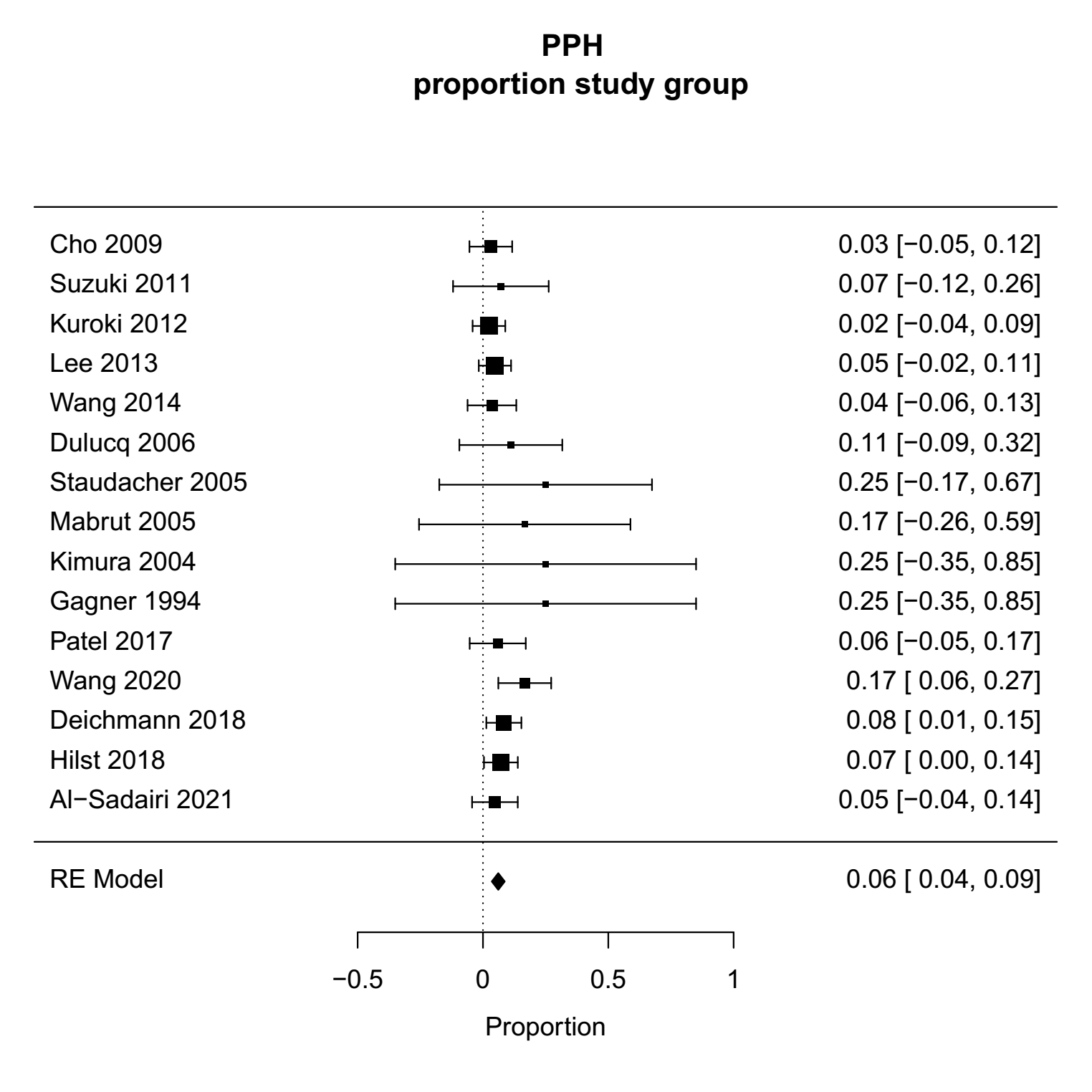


Supplemental Figure 1: Forest plot; postpancreatectomy haemorrhage rate of all HPD`s

# PPH

# OPD HPD


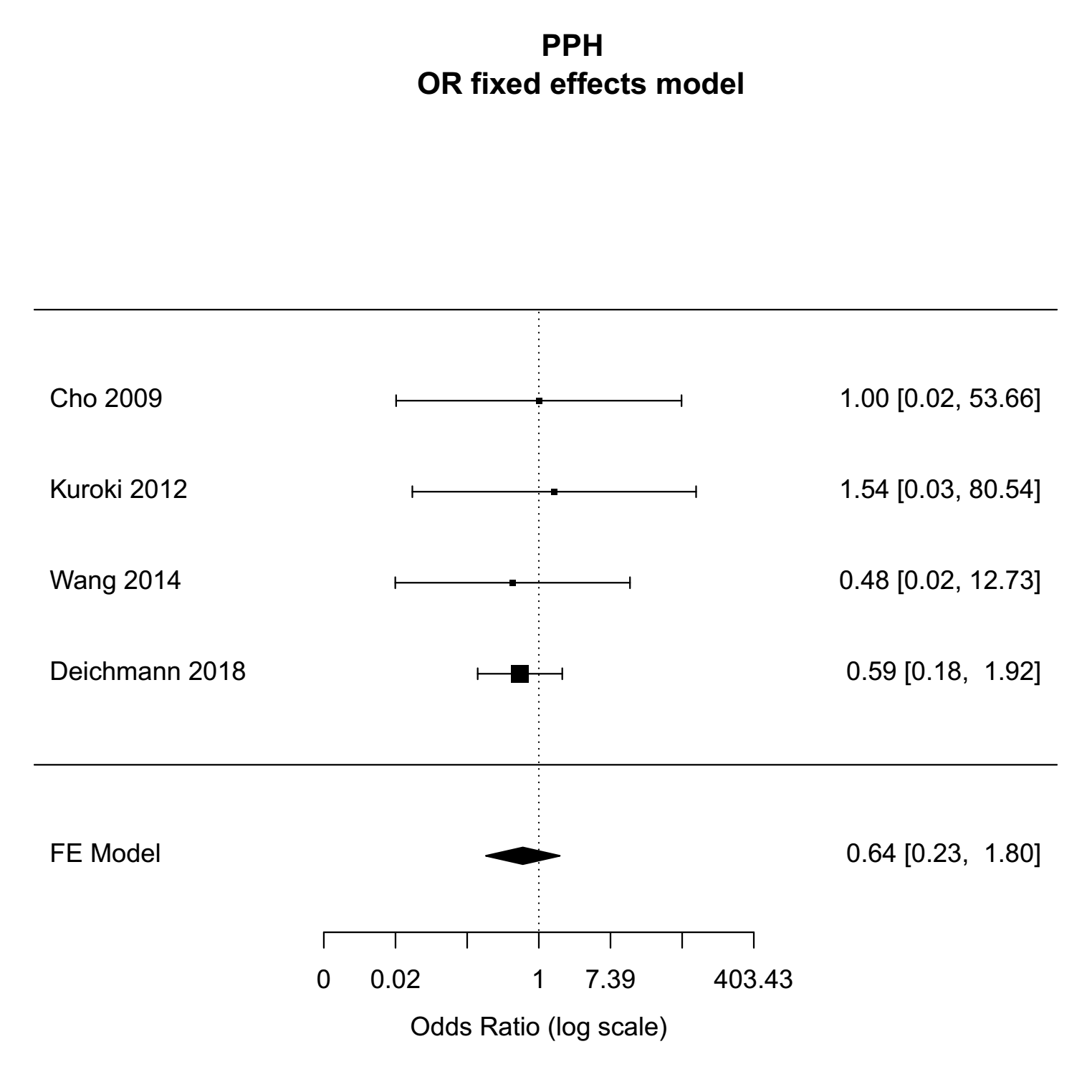


## Supplemental Figure 2: Forest plot; postpancreatectomy haemorrhage rate in comparative studies (comparison between HPD and OPD)

# DGE


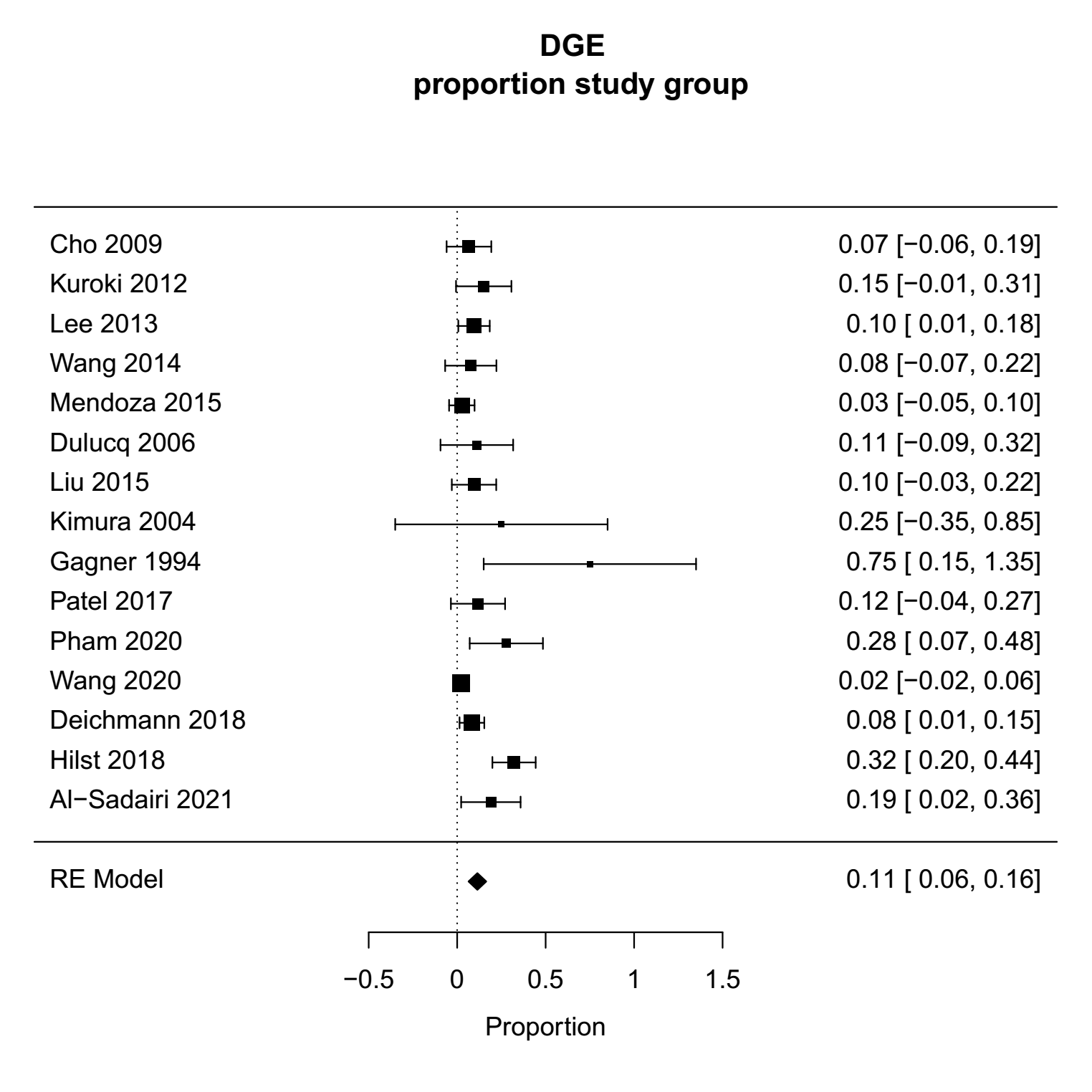


Supplemental Figure 3: Forest plot; delayed gastric emptying rate of all HPD`s

# DGE

OPD HPD


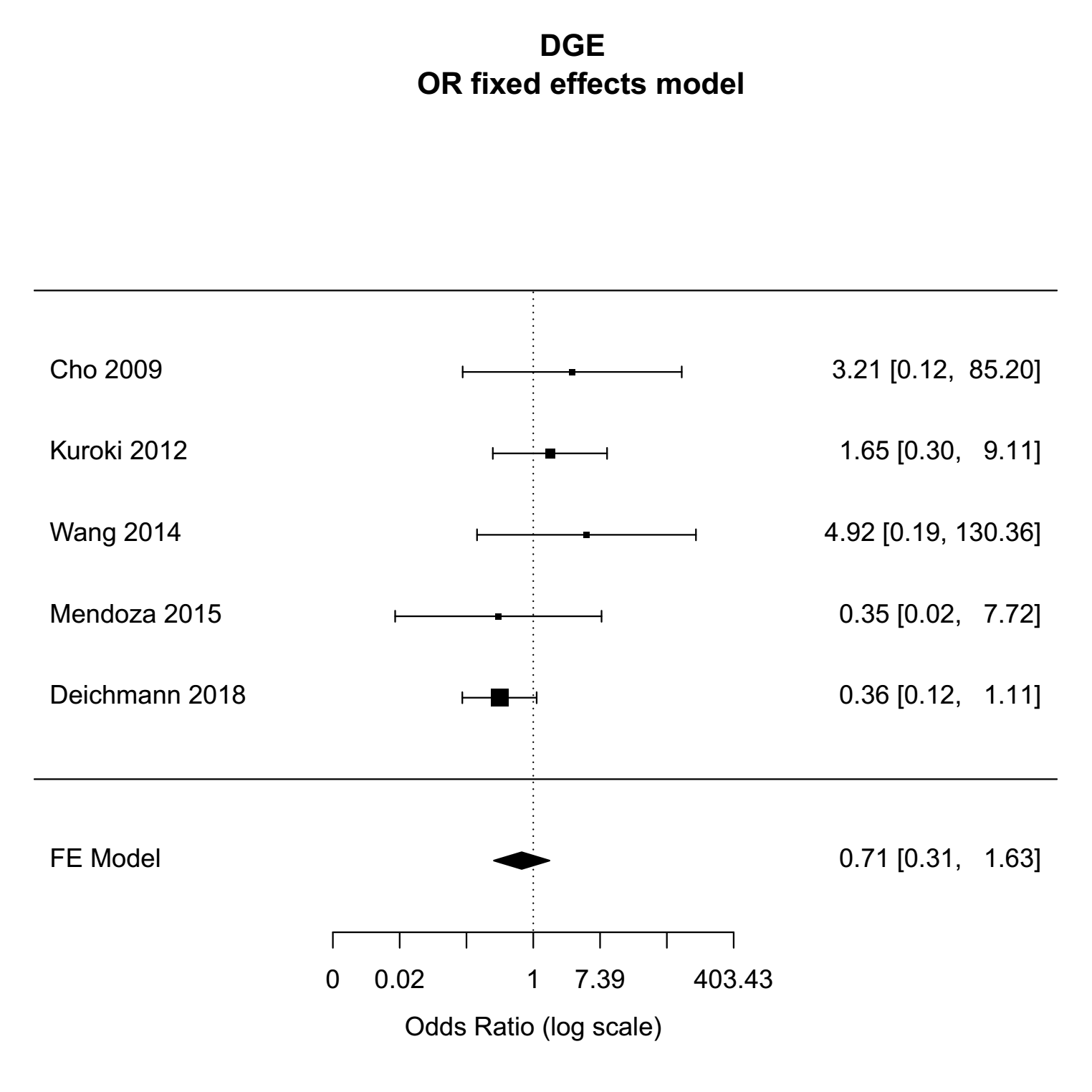


##

Supplemental Figure 4: Forest plot; delayed gastric emptying rate in comparative studies (comparison between HPD and OPD)

# hepaticoenterostomy leakage


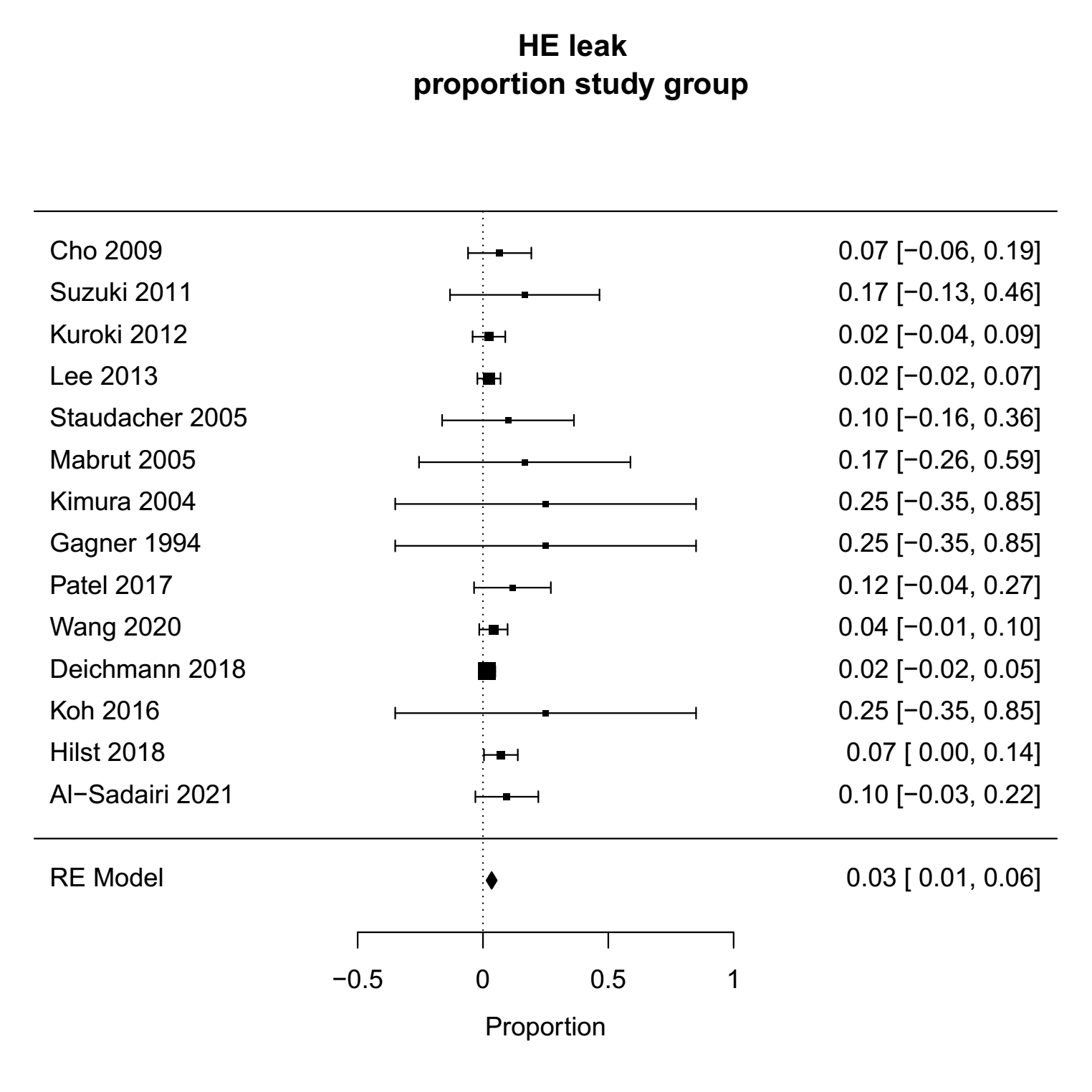


Supplemental Figure 5: Forest plot; hepaticoenterostomy leakage rate of all HPD`s hepaticoenterostomy leakage

hepaticoenterostomy leakage

OPD HPD


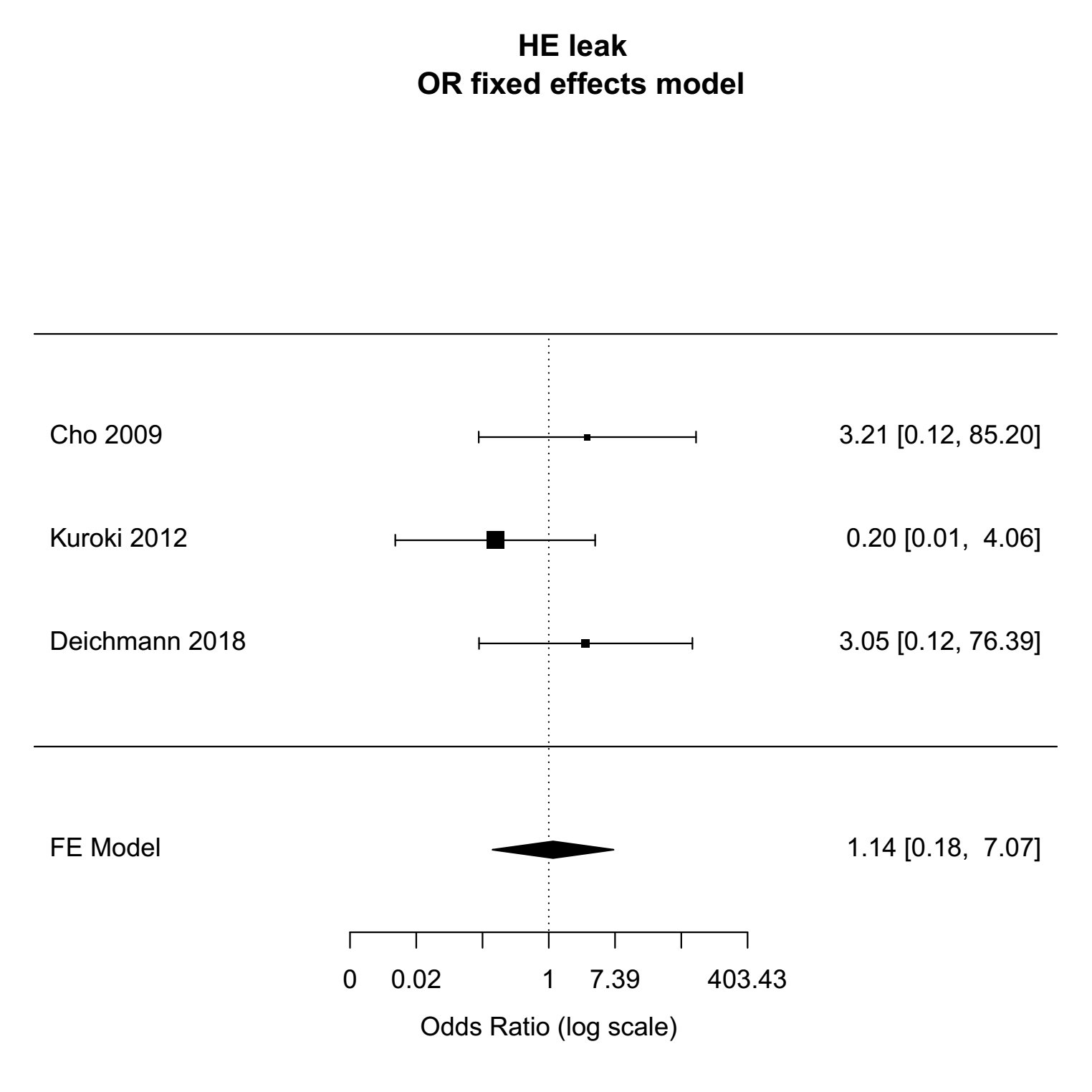


##

Supplemental Figure 6: Forest plot; hepaticoenterostomy lekage in comparative studies (comparison between HPD and OPD)

# morbidity


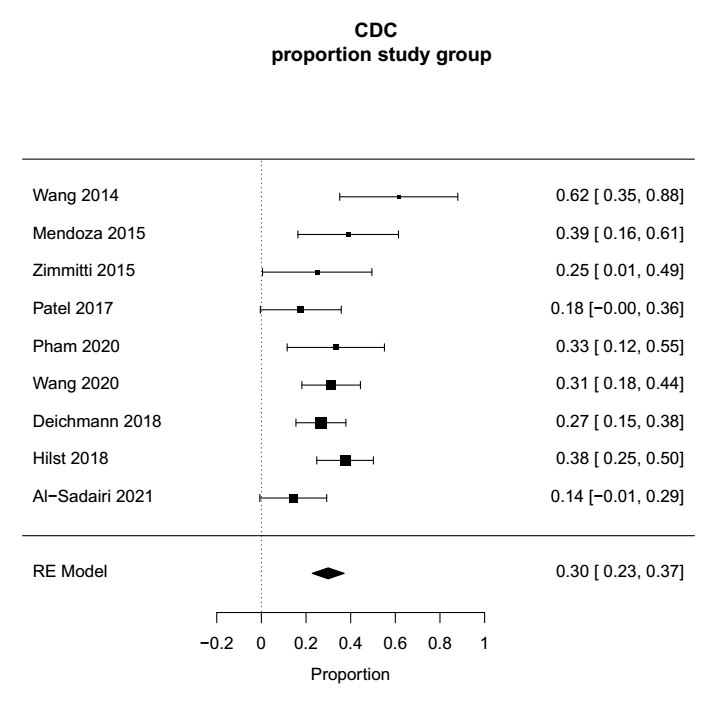


Supplemental Figure 7: Forest plot; Morbidity rate according to Clavien–Dindo 2 – 5 of all HPD`s morbidity

morbidity

OPD HPD


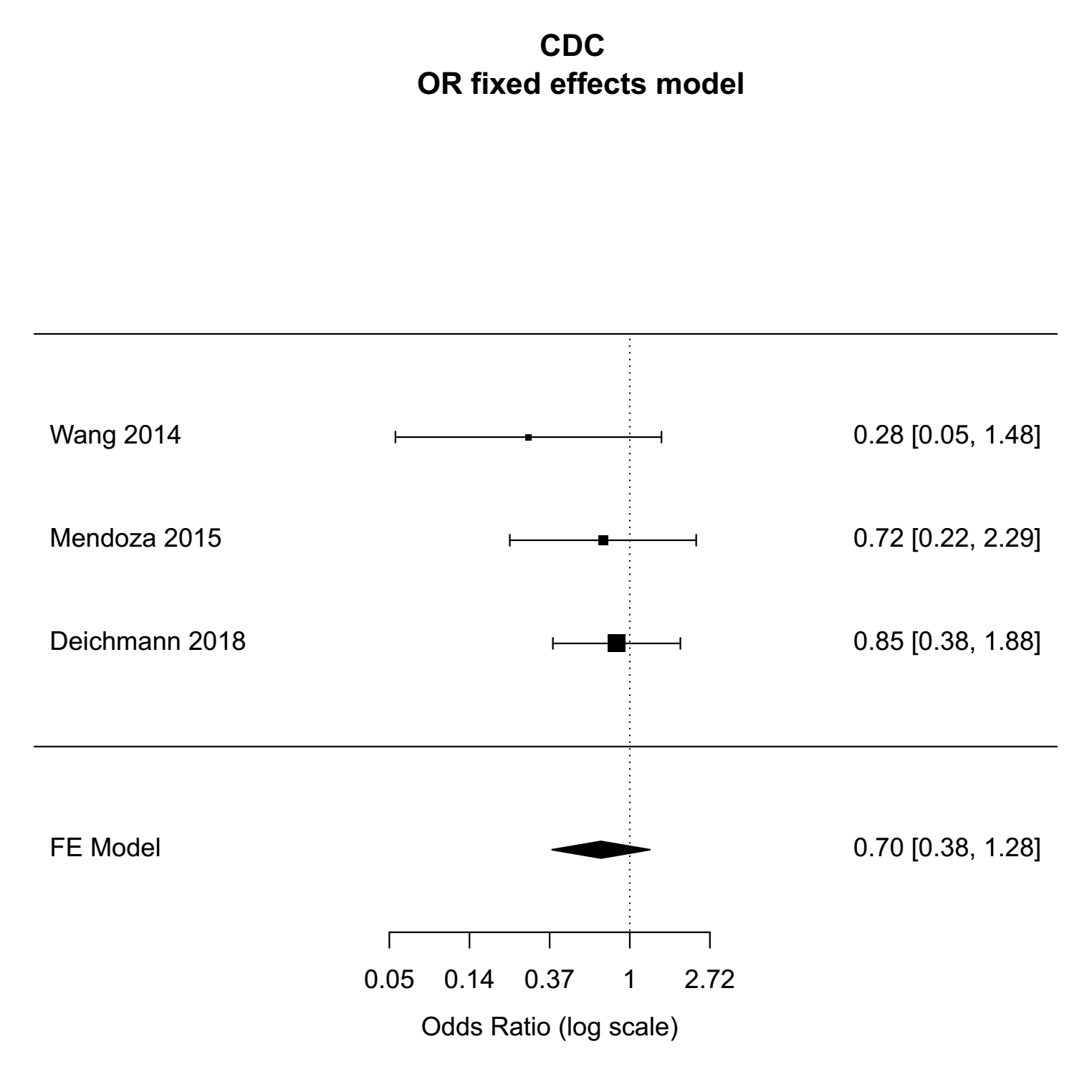


##

Supplemental Figure 8: Forest plot; Morbidity rate according to Clavien–Dindo 2 – 5 in comparative studies (comparison between HPD and OPD)

# complications


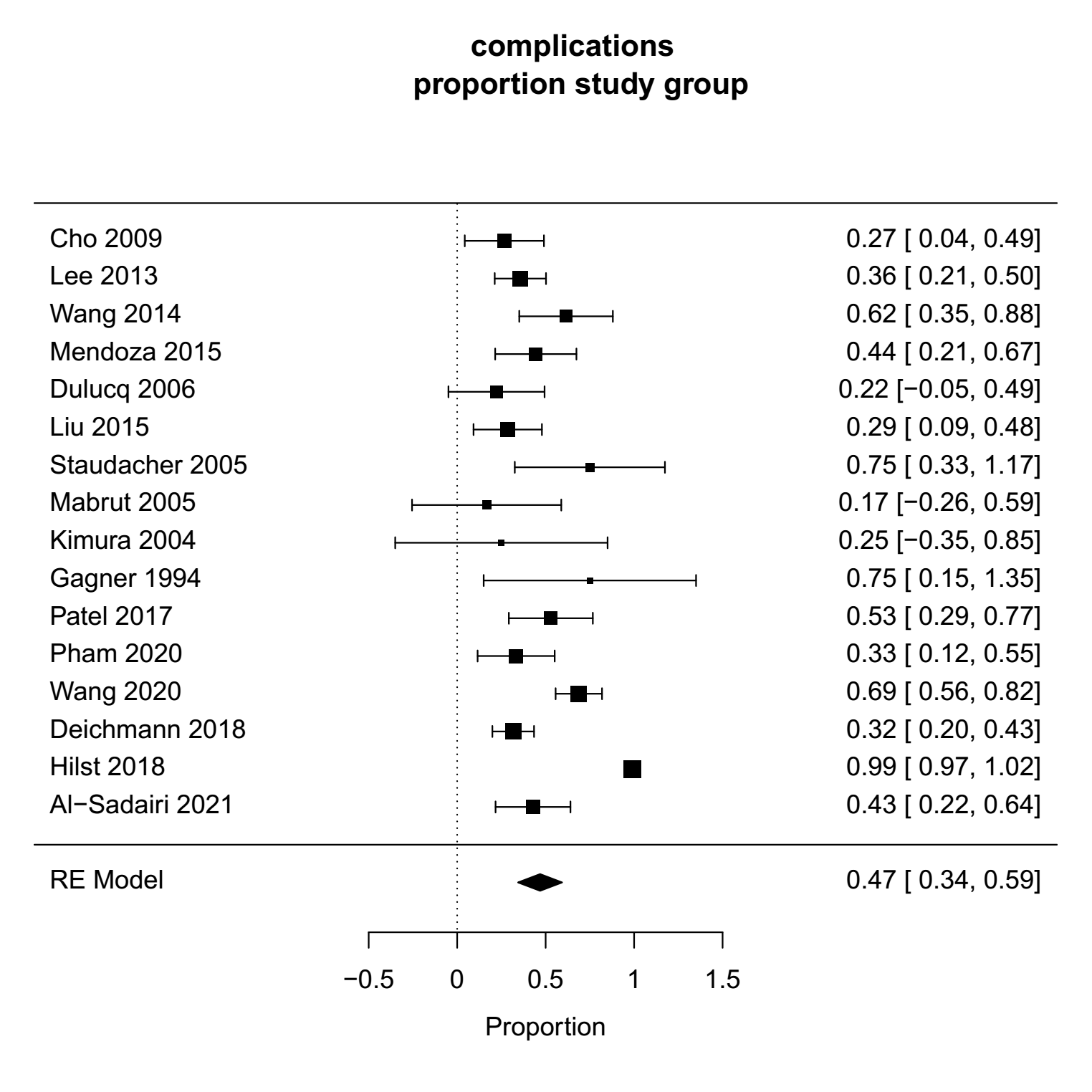


Supplemental Figure 9: Forest plot; overall complications of all HPD`s

# complications

# OPD HPD


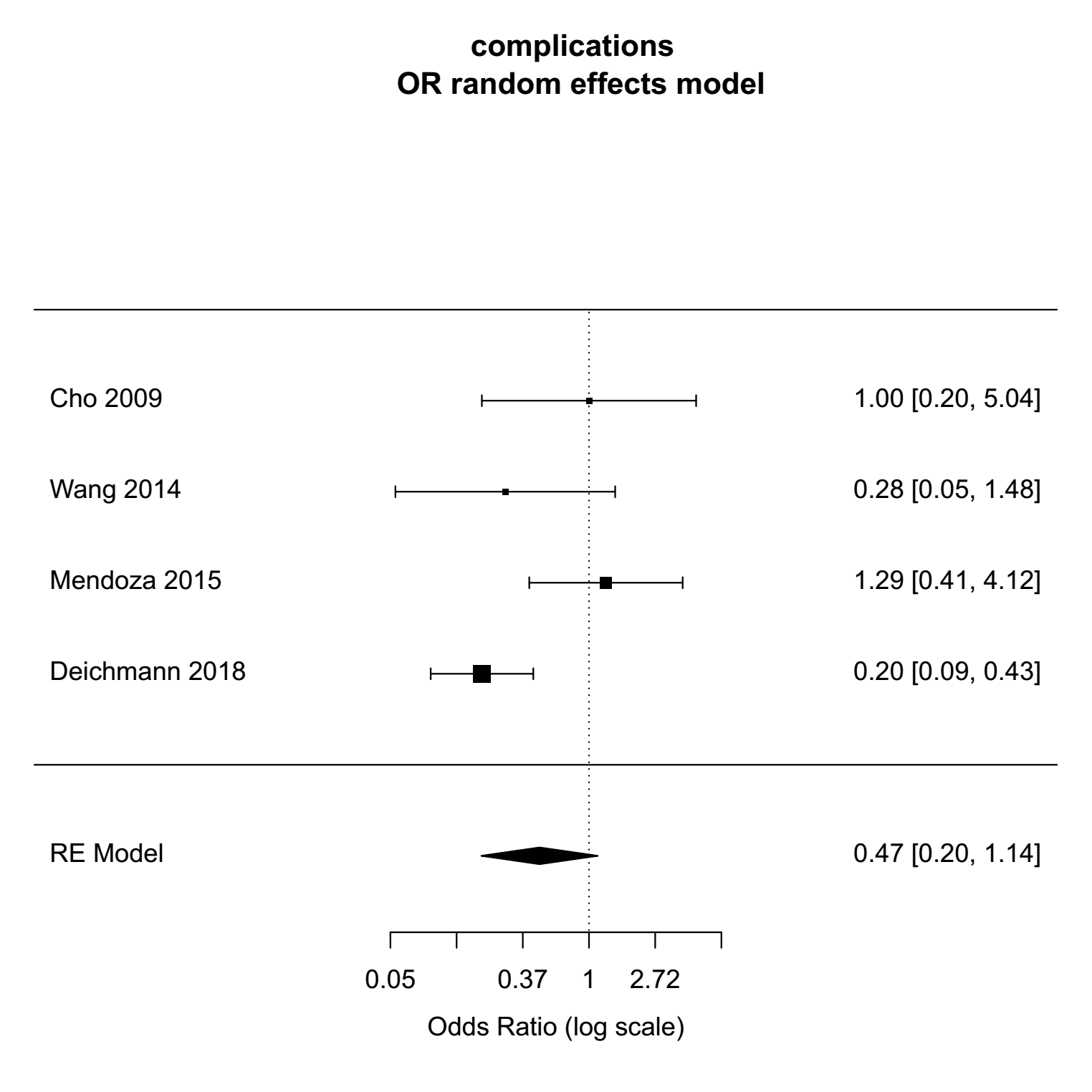


##

Supplemental Figure 10: Forest plot; overall complications rate in comparative studies (comparison between HPD and OPD)

# SSI


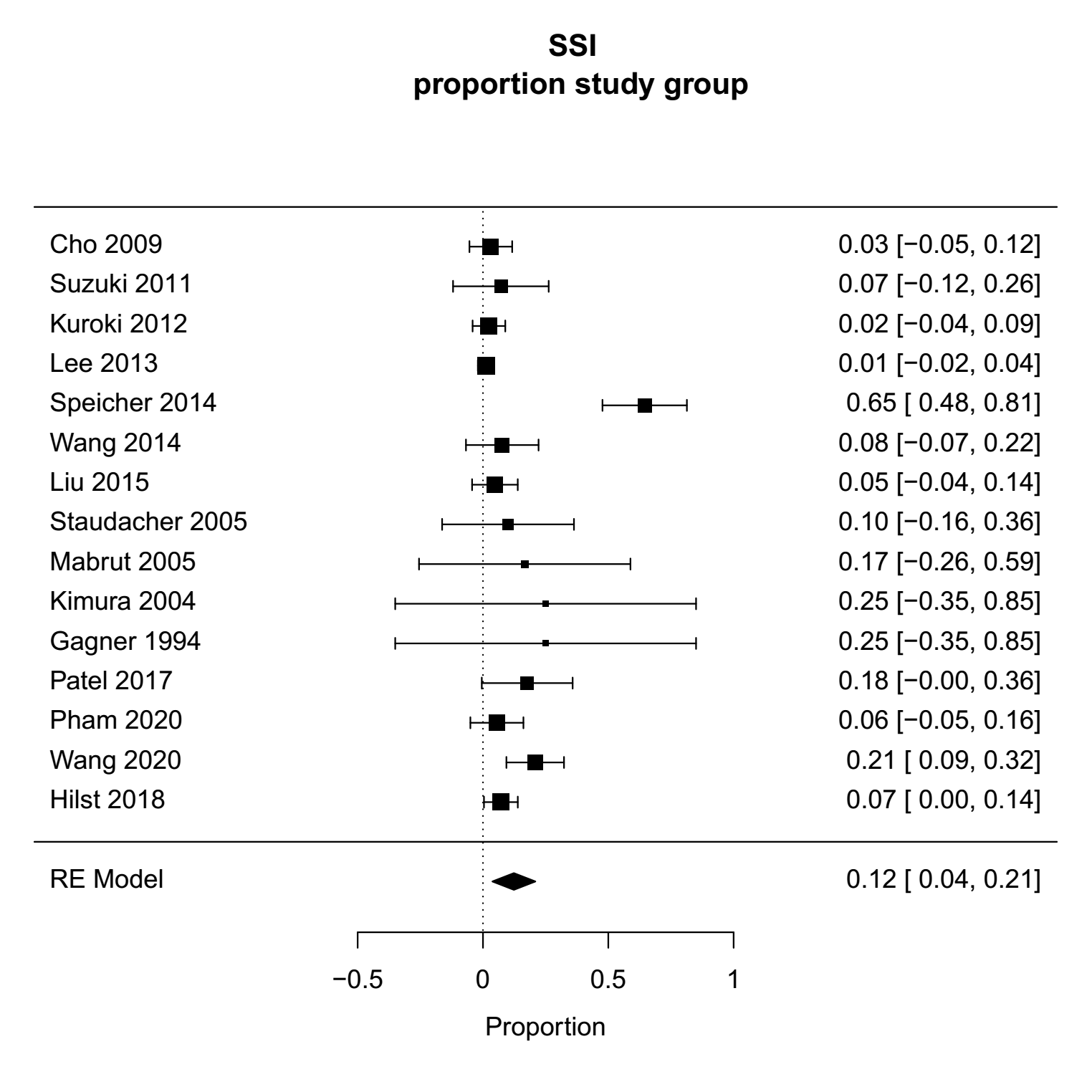


Supplemental Figure 11: Forest plot; surgical site infection rate of all HPD`s

# SSI

OPD HPD


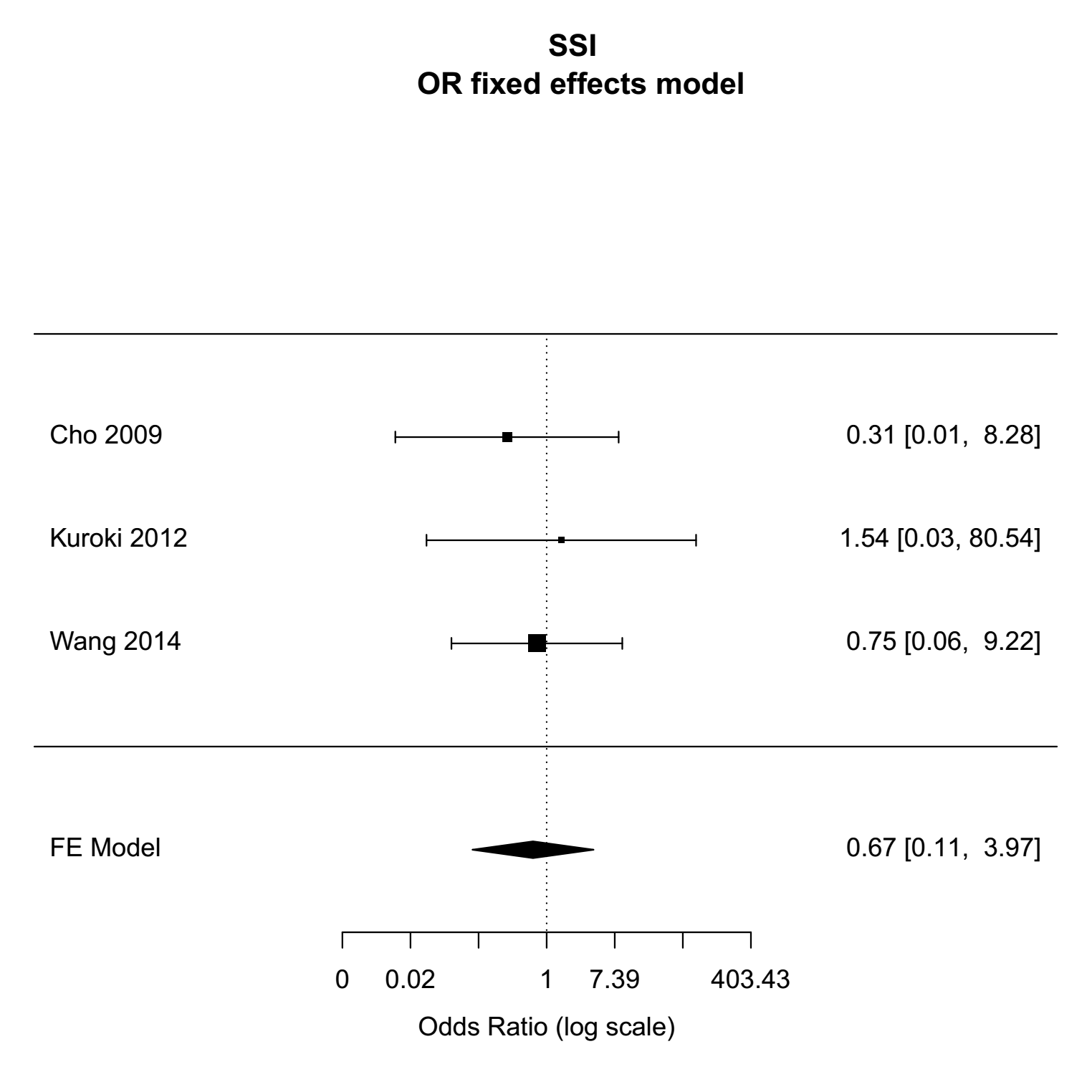


Supplemental Figure 12: Forest plot; surgical site infection rate in comparative studies (comparison between HPD and OPD)

# OHS


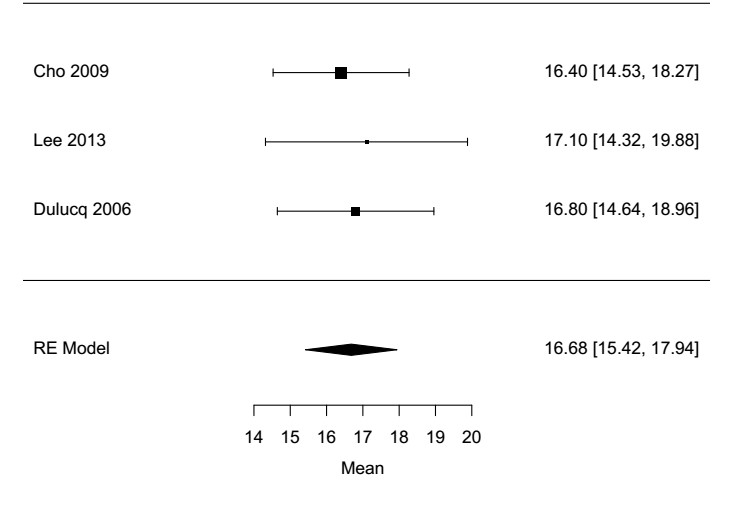


Supplemental Figure 13: Forest plot; overall hospital stay in days of all HPD`s

# OHS

## OPD HPD


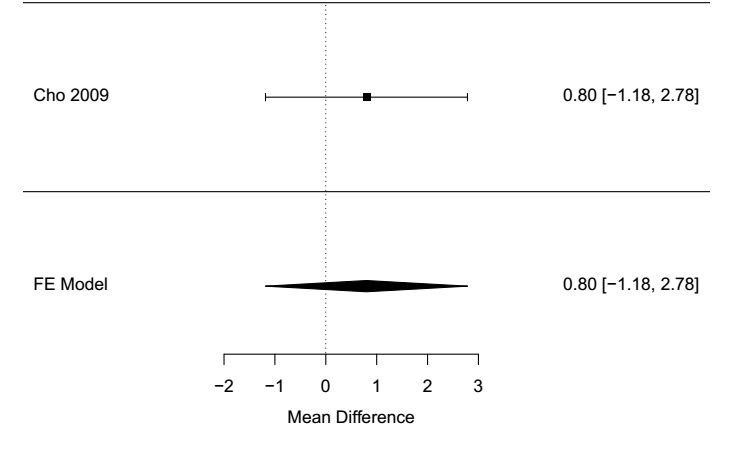


Supplemental Figure 14: Forest plot; mean difference of overall hospital stay between HPD and OPD in days

# Reoperation


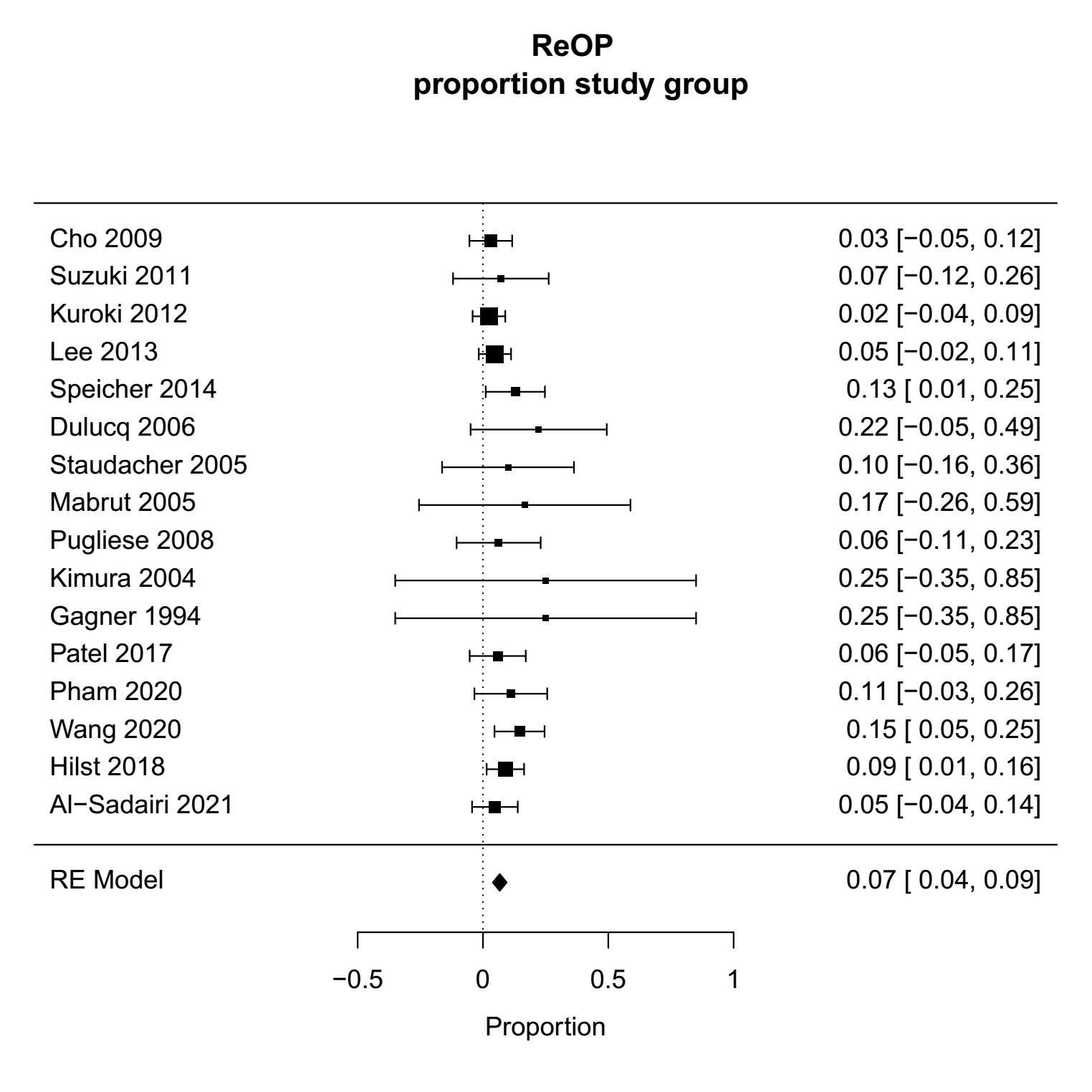


Supplemental Figure 15: Forest plot; reoperation rate of all HPD`s

# Reoperation

# OPD HPD


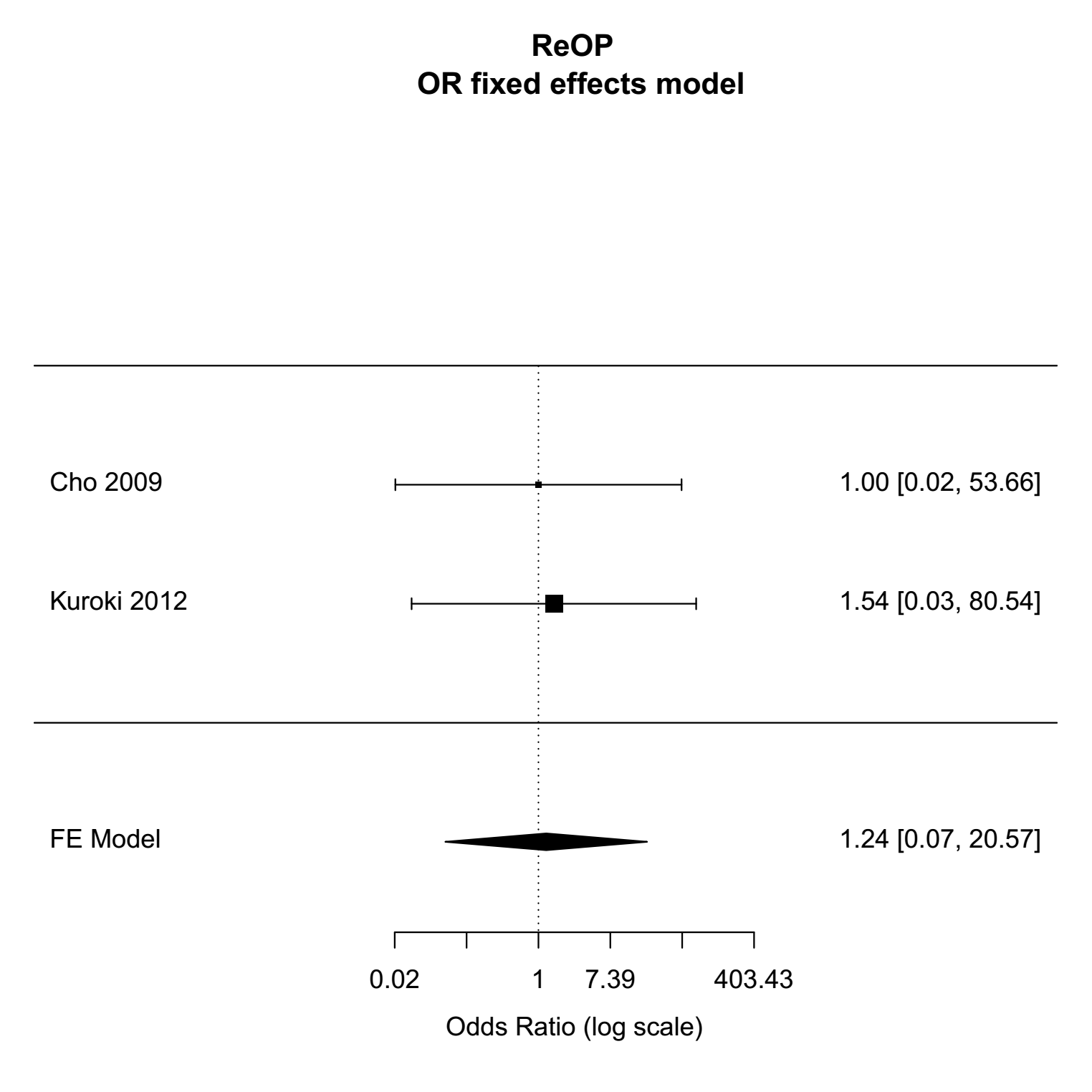


##

Supplemental Figure 16: Forest plot; reoperation rate in comparative studies (comparison between HPD and OPD)
